# A sodium binding system alleviates acute salt stress during seawater acclimation in eels

**DOI:** 10.1186/s40851-017-0081-8

**Published:** 2017-12-12

**Authors:** Marty Kwok Shing Wong, Takehiro Tsukada, Nobuhiro Ogawa, Supriya Pipil, Haruka Ozaki, Yutaka Suzuki, Wataru Iwasaki, Yoshio Takei

**Affiliations:** 1 Atmosphere and Ocean Research Institute, The University of Tokyo, Kashiwa City, Japan; 2 Department of Biomolecular Science, Toho University, Funabashi City, Japan; 3 Department of Biological Sciences, Graduate School of Science, The University of Tokyo, Tokyo, Japan; 4 Department of Computational Biology, Graduate School of Frontier Sciences, The University of Tokyo, Kashiwa City, Japan; 5 Bioinformatics Research Unit, Advanced Center for Computing and Communication, RIKEN, Wako City, Japan

**Keywords:** Sodium storage, Osmoregulation, Mucus, Teleost, Euryhaline, Transcriptome

## Abstract

**Background:**

Teleosts transiting from freshwater (FW) to seawater (SW) environments face an immediate osmotic stress from ion influxes and water loss, but some euryhaline species such as eels can maintain a stable plasma osmolality during early SW exposure. The time course changes in the gene expression, protein abundance, and localization of key ion transporters suggested that the reversal of the ion transport systems was gradual, and we investigate how eels utilize a Na-binding strategy to slow down the ion invasion and complement the transporter-mediated osmoregulation.

**Results:**

Using an electron probe micro-analyzer, we localized bound Na in various eel tissues in response to SW transfer, suggesting that the Na-binding molecules were produced to sequester excess ionic Na^+^ to negate its osmotic potential, thus preventing acute cellular dehydration. Mucus cells were acutely activated in digestive tract, gill, and skin after SW transfer, producing Na-binding molecule-containing mucus layers that fence off high osmolality of SW. Using gel filtration HPLC, some molecules at 18 kDa were found to bind Na in the luminal secretion of esophagus and intestine, and higher binding was associated with SW transfer. Transcriptome and protein interaction results indicated that downregulation of Notch and β-catenin pathways, and dynamic changes in TGFβ pathways in intestine were involved during early SW transition, supporting the observed histological changes on epithelial desquamation and increased mucus production.

**Conclusions:**

The timing for the activation of the Na-binding mechanism to alleviate the adverse osmotic gradient was temporally complementary to the subsequent remodeling of branchial ionocytes and transporting epithelia of the digestive tract. The strategy to manipulate the osmotic potential of Na^+^ by specific binding molecules is similar to the osmotically inactive Na described in human skin and muscle. The Na-binding molecules provide a buffer to tolerate the salinity changes, which is advantageous to the estuary and migrating fishes. Our data pave the way to identify this unknown class of molecules and open a new area of vertebrate osmoregulation research.

**Electronic supplementary material:**

The online version of this article (10.1186/s40851-017-0081-8) contains supplementary material, which is available to authorized users.

## Background

Studies on osmoregulation have focused on the transport of ions to drive water movement and it is generally accepted that ions in the extracellular fluid (ECF) are regulated at a relatively constant level to maintain plasma osmolality (ca. 300-350 mOsm) in most vertebrates. The gill, intestine, and kidney are the major osmoregulatory organs in teleosts and successful acclimation in both freshwater (FW) and seawater (SW) environments depends on the composition of epithelial transporters and channels, and metabolic and structural adjustments in those tissues. Externally, the teleost gill possesses various types of ionocytes that absorb ions in FW and excrete ions in SW [1–3]. Teleosts drink copiously in SW and the intestine absorbs the imbibed water by actively taking up monovalent ions (Na^+^/Cl^−^) and precipitating divalent ions (Ca^2+^, Mg^2+^, SO_4_^2−^) [4–6]. The kidneys perform glomerular filtration to excrete excess water by producing copious diluted urine in FW [7, 8] while those in SW actively secrete divalent ions in the proximal tubules [9–11]. These mechanisms were mostly derived from studies using euryhaline species (e.g. eels, salmon, killifish, etc.) fully acclimated to either FW or SW, whereas knowledge during the early transition phase between salinities is limited.

A literature survey on the time-course changes in plasma ions and osmolality after FW to SW transfer in various teleosts revealed an intriguing phenomenon (Table 1): The time to reach the peak values appears to be immediate and rapid in stenohaline species but delayed in some euryhaline species. Goldfish is a stenohaline species and SW transfer increased the plasma Na^+^ rapidly to lethal levels with 30 min [12]. Stream resident stickleback is a FW stenohaline population, and SW transfer increased the plasma Na^+^ within 12 h to lethal concentrations, while euryhaline anadromous population could maintain a stable plasma Na^+^ [13]. Japanese medaka is a moderate euryhaline species and FW medaka can acclimate to 2/3 SW directly with a significant increase in plasma Na^+^ within 3 h after the transfer (Table 1).

**Table 1:** Effects of salinity transfer on the levels of plasma Na^+^, Cl^−^, and osmolality in common teleost models

Species (Common name)	Salinity Transfer (‰)	Time to reach maximum change in plasma (c.a. change from pre-transfer values)			References
		Na^+^	Cl^−^	Osmolality	
*Anguilla japonica* (Japanese eel)	0 → 35‰	3 d (144 → 223 mM)	N/A	N/A	[14]
	0 → 35‰	12 h (145 → 190 mM)	N/A	12 h (270 → 365 mOsm)	[15]
	0 → 35‰	24 h (152 → 209 mM)	3 d (100 → 168 mM)	3 d (340 → 457 mOsm)	[16]
*Anguilla anguilla* (European eel)	FW → SW	2 d (153 → 210 mM)	2 d (83 → 165 mM)	N/A	[17]
*Oryzias latipes* (Japanese medaka, HdrR)	0 → 25‰	3 h (145 → 178 mM)	N/A	N/A	Present study
*Oreochromis mossambicus* (Mozambique tilapia)	0 → 25‰	24 h (150 → 190 mM)	12 h (140 → 175 mM)	12 h (320 → 400 mOsm)	[55]
	0 → 20‰	12 h (140 → 220 mM)	12 h (130 → 220 mM)	12 h (275 → 410 mOsm)	[56]
	0 → 15‰	N/A	N/A	24 h (335 → 395 mOsm)	[57]
*Oreochromis niloticus* (Nile tilapia)	0 → 15‰	N/A	12 h (140 → 200 mM)	12 h (325 → 430 mOsm)	[58]
	0 → 15‰	N/A	N/A	24 h (315 → 395 mOsm)	[57]
*Gasterosteus aculeatus* (Three-spined stickleback)					[13]
anadromous	3.5 → 35‰	24 h (170 → 200 mM)	N/A	N/A	
stream-resident	3.5 → 35‰	^a^12 h (150 → 250 mM)	N/A	N/A	
*Salvelinus namaycush* (Lake trout)	0 → 30‰	7 d (160 → 220 mM)	24 h (140 → 190 mM)	N/A	[1]
*Salvelinus fontinalis* (Brook trout)	0 → 30‰	24 h (150 → 220 mM)	24 h (130 → 200 mM)	N/A	[1]
*Salmo salar* (Atlantic salmon)	0 → 30‰	24 h (150 → 160 mM)	N.S. (140 → 145 mM)	N/A	[1]
	FW → SW	N/A	24 h (140 → 175 mM)	N/A	[59]
*Fundulus heteroclitus *(Killifish)	FW → SW	8 h (190 → 225 mM)	N/A	N/A	[60]
	FW → SW	24 h (170 → 260 mM)	N/A	24 h (250 → 360 mOsm)	[61]
*Carassius auratus* (Goldfish)	0 → 34‰	^a^25 min (155 → 175 mM)	^a^25 min (104 → 144 mM)	^a^25 min (273 → 359 mOsm)	[12]

*N.S.* No significance, *N/A* Not available

^a^Stenohaline species/strain died after the last sampling time points

**Fig. 1:**
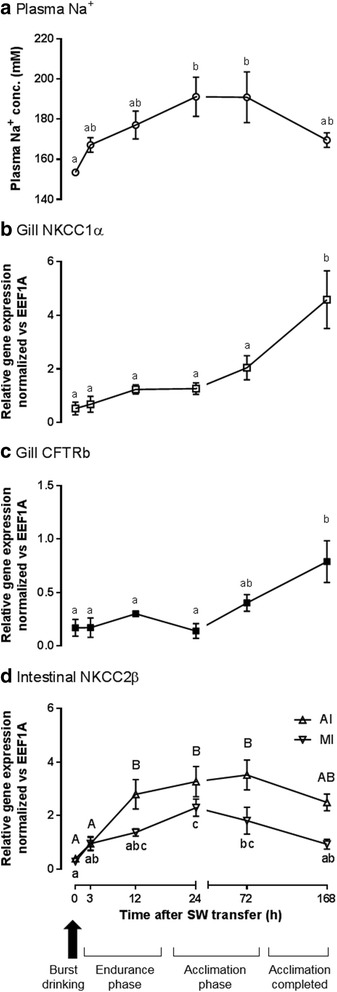
Effects of time-course freshwater (FW) to seawater (SW) transfer on (**a**) plasma Na^+^ concentration (*N* = 6); (**b**) NKCC1α expression in gill; (**c**) NKCC2β expression in anterior (AI) and middle (MI) intestine of eels (*N* = 5). During initial transfer, eels drink SW copiously (arrow) and expose the gill to SW. Plasma Na^+^ was not increased suddenly (endurance phase), subsequently increased during the acclimation period, and gradually established to a new baseline of lower level after SW 7 d (acclimation completed). Similar delayed transient increase was found among NKCC2β expression in the intestines while branchial NKCC1α and CFTRb expression increased significantly after SW 7 d. Statistical differences among different time are indicated by alphabet letters (*p* < 0.05) after one way ANOVA, Tukey’s test

**Fig. 2:**
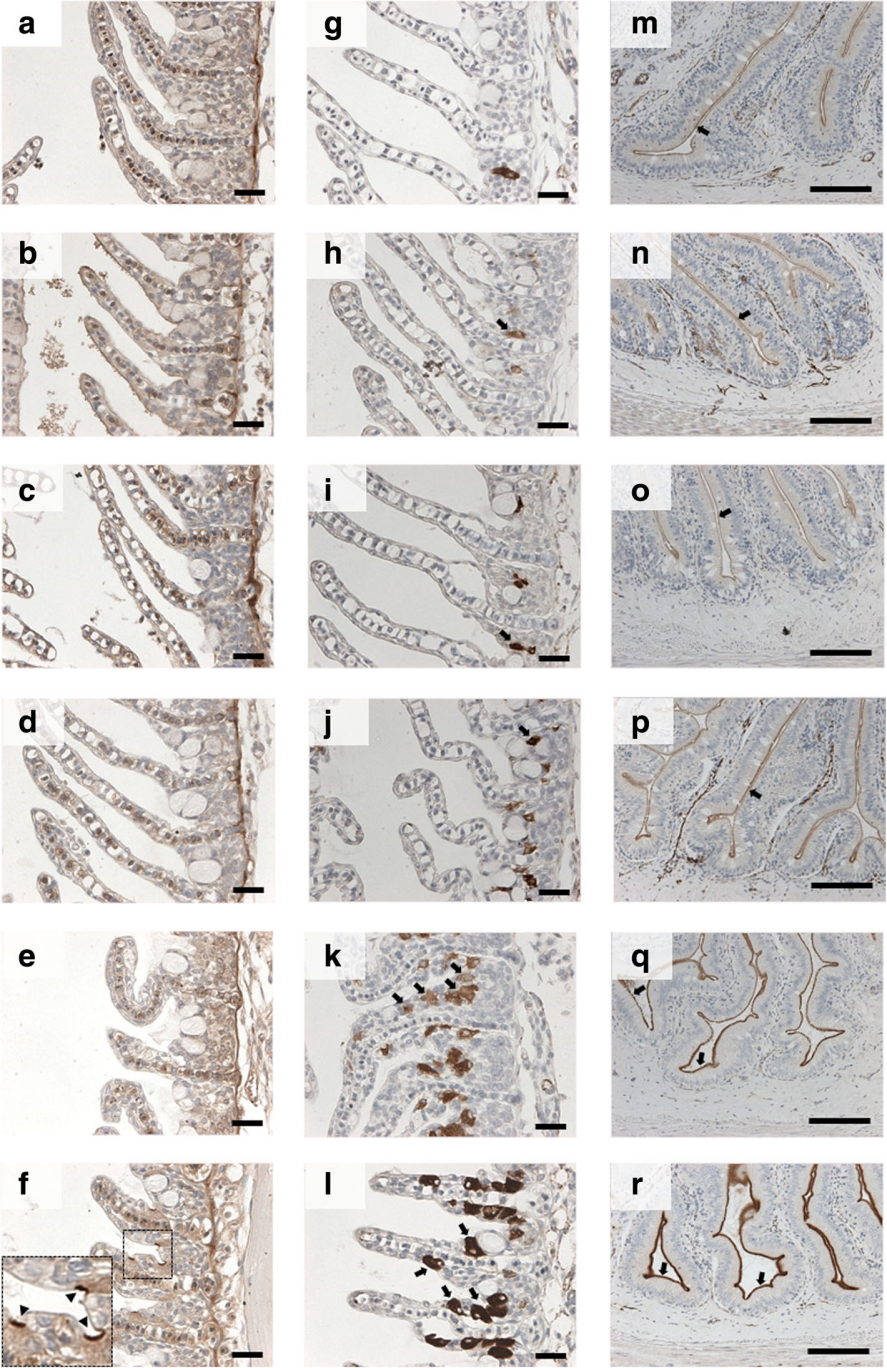
Immunohistochemistry of NKCC and CFTR in the gill and anterior intestine (AI) of eels in FW and time course SW exposure. Apical CFTR signals (arrow heads) on the ionocytes at the interlamellar region are not observed from FW to SW 3 d (**a-e**), and become apparent on the ionocytes of SW 7 d eels (**f**). Basolateral NKCC (arrows) in ionocytes are scarcely observed from FW to SW 1 d (**g-j**), and become increasingly abundant in numbers and intensity from SW 3 d to 7 d (**k**-**l**). Apical NKCC (arrows) on the intestinal epithelia are present in all time course samples (**m**-**r**), but an increasing intensity is observed in SW 3 d to 7 d. Scale bar = 100 μm

When euryhaline eels were transferred from FW to SW, plasma Na^+^ followed a reproducible pattern where it remained unchanged for 6 h, significantly increased after 8–12 h, reached a maximum after 1–3 d, and declined to a new baseline approximately 7 d after the transfer [14–17]. Eels start drinking SW within a minute after transfer from FW [18] and it was suggested that the imbibed SW was desalinated in the esophagus and absorbed by the intestine to compensate for osmotic loss of water in the hyperosmotic environment [6]. However, it was demonstrated that the FW eel esophagus was impermeable to ions and incapable of desalinating imbibed SW [19], suggesting that the initial imbibition is processed by an alternative mechanism other than the established desalination mechanism in SW acclimated eels. The upregulation of Na^+^/K^+^-ATPase (NKA) expression in the gill, intestine, and kidney, did not occur until at least 1 d and reached maximum expression increase after 3–7 d in SW [20]. Similarly, the expression and protein contents of branchial cystic fibrosis transmembrane conductance regulator (CFTR), Na^+^-K^+^-Cl^−^ cotransporter (NKCC) for ion excretion, and intestinal apical NKCC for ion absorption, did not increase soon after SW transfer, nor did the ion transporters being reallocated immediately following the transfer (Figs. 1 and 2). Thus, how can eels maintain their plasma ion levels for the initial hours following SW transfer [17]? A sudden influx of ions into the body from the gill and digestive tract should increase the ion concentration of ECF concurrently, as often observed in stenohaline species or sub-species populations (Table 1). Furthermore, if the ion-transporter systems could effectively remove the influx of ions during early SW transfer, there should not be a need to upregulate the ion-transporter expressions 1–3 d after the transfer and we should not observe any significant changes in the plasma ions and osmolality. Therefore, the gradual rise in plasma Na^+^ in euryhaline species following SW transfer suggests that the increase in plasma ion concentration was slowed down by some unknown mechanisms. This phenomenon is irrespective to the size of fish as small fish models including killifish and marine stickleback, which possess a large surface area to volume ratio, also displayed such delayed increase in plasma Na^+^.

Recently, it was suggested that mammalian skin and muscle store Na in an osmotically inactive form and high contents were significantly related to hypertension [21]. Using magnetic resonance imagining, a large amount of Na storage was demonstrated in the skin and muscle in humans, which was independent of tissue water content [22]. The stored Na could be mobilized and utilized in growth under low salt diet conditions in mammals [23]. Besides hypertension, the high Na levels in the skin and muscle were also correlated to bacterial and parasite infections, and the effect was dependent on macrophage expression of *NFAT5* [24]. Glycosaminoglycans (GAGs) with high negative charges in muscle and skin were suggested to be involved in the immobilization of ionic Na^+^ to store Na in the skin [23, 25]. The growing evidence of osmotically-inactive Na storage supports the existence of Na-binding molecules in vertebrates and they could have been utilized in euryhaline fishes to slow down Na^+^ entry during the initial phase of SW acclimation.

In the present study, we aimed to demonstrate whether similar Na storage occurs in eels, and how this osmotically-inactive Na is correlated to their supreme euryhalinity of this species. We hypothesized that most euryhaline teleosts including eels utilize binding systems to manipulate plasma Na^+^ concentrations during the initial phase of SW transfer, facilitating short-term homeostasis in body fluid composition. However, this mechanism could be limited or saturated within hours, resulting in an inevitable increase in plasma ion concentrations. The capacity to tolerate changes in ion fluxes before the maturation of new transporting epithelia in various osmoregulatory tissues could be one factor that determines the species acclimation abilities under fluctuating salinities.

## Methods

### Animals

Juvenile Japanese eel (*Anguilla japonica*) and medaka (*Oryzias latipes*, HdrR strain) were maintained according to [16, 26] respectively. All animal studies were performed according to the Guideline for Care and Use of Animals approved by the Animal Experiment Committee of the University of Tokyo.

### Experimental setup, sampling, and measurement of Na

FW eels were transferred to SW in a time course according to procedures described in [16]. The gill, skeletal muscle, and whole digestive tract of eels were dissected out and rinsed in 0.9% NaCl saline solution to remove seawater attached on the surface. The digestive tract was further flushed with saline to remove the content inside and divided into esophagus, stomach, anterior intestine, middle intestine, posterior intestine, and rectum. The tissues were plotted dried, weighed, and then dried overnight in an oven at 120 °C. Dried tissues were weighed and homogenized in 0.1 M HNO_3_ (1: 10 dry weight: volume) by a bean cell disruptor (Micro Homogenizing System Micro Smash KLV-MS-100, TOMY, Tokyo, Japan). Aqueous layer was obtained by centrifugation at 10,000 g for 10 min at 4 °C. To increase the number of species in the comparison of euryhalinity, Japanese medaka was challenged with hyperosmotic transfer as no previous study has reported the early plasma Na^+^ profile in medaka. FW medaka were transferred to 2/3 SW (25‰) for 1 h, 3 h, 1 d, and 7 d (*N* = 5–8). Pre-transfer medaka were taken as FW intact control. Anesthetized medaka individuals were punctured at the caudal vessel using needle-shaped heparinized micro-capillaries to collect blood samples. The blood samples were centrifuged in a hematocrit centrifuge at 10,000 rpm for 5 min at 25 °C to obtain plasma for Na^+^ measurement. Concentrations of Na^+^ in plasma and tissue extracts were measured by the atomic absorption spectrophotometer described previously [16].

### Histology of eel tissues

Eel digestive tract, muscle, and gill were fixed in 4% paraformaldehyde in 0.1 M phosphate buffer at pH 7.4 for 1 d at 4 °C. The tissues were then transferred to 70% ethanol for storage at 4 °C until further processing. Fixed tissues were dehydrated in serial alcohol/xylene solutions and embedded in Paraplast wax (Leica Biosystems, Nussloch, Germany). Cross sections (4 μm) were prepared and mounted on MAS-coated microscope slides (Matsunami, Osaka, Japan) for histology. The tissue sections were de-paraffinized by xylene and rehydrated to deionized water by serial alcohol solutions. For PAS staining, the sections were incubated in 0.5% periodic acid for 10 min followed by Schiff’s reagent (Wako Pure Chemical, Osaka, Japan) for 15 min. After washing in sulfurous acid solution three times, the stained sections were counterstained with hematoxylin. For Alcian blue, sections were incubated in Alcian blue (pH 2.5) or (pH 1.0) for 30 min, rinse in 3% acetic acid (in pH 2.5) or 0.1 M HCl (in pH 1.0), and counterstained with nuclear fast red. For immunohistochemistry, the hydrated sections were treated with 0.2% H_2_O_2_ in methanol for 30 min to inactivate endogenous peroxidase activity and then non-specific sites were blocked by 2% normal goat serum (NGS) in PBS (pH 7.4) for 60 min. Monoclonal CFTR (MAB25031, R&D Systems, Minneapolis, MN, USA) and NKCC (T4, DSHB, IA, USA) antibodies were diluted in 1:300 and 1:10,000 respectively in PBS containing 2% NGS and 0.01% NaN_3_ and incubated with the sections at 4 °C for 16–18 h in a moist chamber saturated with water vapor. Immunoreactive signals were developed using a Vectastain ABC Elite kit (Vector Laboratories, CA, USA) and 3,3′-diaminobenzidine (DAB) as the color reagent according to manufacturer’s protocols. Sections were counterstained with hematoxylin after DAB color development. Sections were observed using a BX-63 microscope (Olympus, Tokyo, Japan). Oval shape cells on the epithelia with strong PAS- and Alcian blue-stainings are considered as mucus cells.

### Na localization by electron probe micro-analyzer (EPMA)

Eel tissues including muscle, gill, esophagus, anterior intestine, and middle intestine were snap frozen in liquid propane (−180 °C), and fixed in 2% osmium tetroxide in acetone for 3 d at −80 °C. The samples were subsequently thawed in a sequence of −20 °C for 2 h, 4 °C for 2 h, and 25 °C for 1 h. Tissues were then washed 3 times in acetone and embedded in EPON812 resin (Electron Microscopy Sciences, Hatfield, PA, USA). The tissue blocks were trimmed and polished with glass knives to obtain a flat surface of cross-section. The surfaces were coated with Pt-Pd and analyzed with a JXA-8230 EPMA (JEOL, Tokyo, Japan). Backscattered-electron imaging was used to detect the fine structure of specimens. Element distribution images (X-ray intensity maps) were obtained under an accelerating voltage of 15 kV, with probe current and diameter at 50 nA and 5 μm respectively. The Ka lines of Na and Cl, Ma lines of Os were measured simultaneously by three wavelength-dispersive spectrometers. ZAF matrix corrections were used to obtain the quantitative maps for Na. Quantitative analysis for Na was performed using albite (NaAlSi3O8) as the Na standard and internal standard data were used for other elements. For qualitative analysis on individual spots, the probe diameter was set to 1 μm with 50 nA probe current.

### Partial purification of Na-binding molecules from esophageal and intestinal secretion using size exclusion chromatography

Since we discovered the Na-binding molecules could be rich in the mucus of esophagus and intestine from histology and EPMA data, we attempted to partially purify the molecules using gel filtration chromatography, exploiting Na-binding characteristics of the molecules in the detection system. The esophagus and intestine of eels from FW and SW 3 h (*n* = 5) were dissected out without damaging the integrity of the luminal contents. The eels were fasted and no food residue was observed. Using a 10 mL syringe attached to an 18 G blunt-end needle, the content of esophagus and intestine was washed into a pre-chilled 15 mL tube with 10 mL ice-cold distilled water. The remaining luminal content was further decanted with gentle squeezing with forceps to extract surface mucus. The extracted luminal secretions were frozen at −80 °C, lyophilized, and stored at −20 °C until further analysis.

The lyophilized luminal secretions were reconstituted in 1 mL 0.1 M Tris-HCl (pH 7.4) and centrifuged at 20,000 g for 5 min at 25 °C to remove debris. The supernatants were injected into a HPLC system to resolve the contents according to the molecular size using a gel filtration column (Biofine GFC PO-4 K, Japan Spectroscopic, 300 mm × 7.5 mm ID, exclusion limit 4 kDa) equilibrated with 0.1 M Tris-HCl (pH 7.4) at a flowrate of 0.5 mL/min. The gel filtration system were calibrated with a gel filtration standard kit (BioRad Laboratories, Hercules, CA, USA) to determine the molecular size of the elution. Fractions at 2 min intervals (1 mL) were collected by a fraction collector and lyophilized. The freeze-dried fractions were reconstituted in 1 mL 0.1 M HCl and incubated at 55 °C overnight. The acidified fractions (10 μL) were diluted in 2.5 mL distilled water and the Na contents were measured by the atomic absorption spectrophotometer. Total protein contents in the fractions were measured by Bradford method using BioRad protein assay dye reagent (Bio-Rad Laboratories, Hercules, CA, USA). Since the extraction and purification system did not contain additional Na, Na contents in the fractions other than the flow-through are considered as molecule-bound Na from the luminal secretions of esophagus and intestine, while Na contents in the flow-through fractions are considered as free ionic Na^+^. Total bound Na in the secretion is defined as the sum of Na contents in the non-flow-through fractions.

### Real-time PCR of known marker genes for ion transport and mucus secretion

Quantitative PCR were performed according to [19, 27]. Real-time PCR primer sequences were as follow: *CFTR*b (F: CCCTCATCCTGTTCGATTTGGT; R: TGAAGATGCACGGCCTCATTATG), *SLC12A1*b (NKCC2β) (F: TGTGTGATGATGTCCGCTG; R: CAAGACGTGCTGAGTGTCATA), *SLC12A2*a (NKCC1α) (F: AGCGATACAGAAAGACGAC; R: CTTCTTCTGGAACTGCTGAC), *MUC2* (F: ACGTTTGTGTGGAGAAGGAG; R: TCGCAGTACGTCATGTCAATC), *MUC5AC* (F: CTTTGAGCTTCGCCTGTCT; R: CAGCTCTGTCCCAGCAAATA), and *EEF1A* (F: CTGAAGCCTGGTATGGTGGT; R: ACGACGGATTTCCTTGACAG).

### Transcriptome of gill, anterior, and posterior intestine by RNA-seq

The total RNAs of eel gill (FW 1 h [*n* = 3], SW 1 h [*n* = 3], FW 3 h [*n* = 3], SW 3 h [*n* = 3]), anterior (FW 1 h [*n* = 3], SW 1 h [*n* = 2], FW 3 h [*n* = 3], SW 3 h [*n* = 2]) and posterior intestines (FW 1 h [*n* = 3], SW 1 h [*n* = 5], FW 3 h [*n* = 3], SW 3 h [*n* = 5]) were extracted using Isogen, reverse transcribed to cDNA libraries according to [26]. For eel RNA-seq, the sequenced reads were mapped to the Japanese eel genome [28] using TopHat (version 2.0.9) [29]. The mapped reads were pooled for each condition, and genome-guided transcriptome assembly was performed to reconstruct the eel transcripts using Cuffilink version 2.1.1. The assembled transcripts were merged using Cuffmerge, and the merged transcripts were used for quantifying gene expression levels.

For each eel transcript, open reading frames (ORFs) were predicted using EMBOSS getorf (version 6.6.0) [30] with the parameter “-minsize 300”. Then, for each gene, the longest ORF among ORFs predicted from all transcripts belonging to the gene was selected, and the translated amino acid sequence of the ORF was used for the following blast search. The reciprocal blast search was performed using amino acid sequences of medaka and eel using BLASTP in NCBI-BLAST+ (version 2.2.29+) [31] with the parameter “-evalue 1e-5”. Longest amino acid sequence for each medaka gene in Ensembl annotation (release 74) was used. Then, the reciprocal BLAST best hits (RBBH) in terms of E-value were defined as RBBHs between medaka and eel. Gene annotation of eel was guided using the medaka genome as reference database with RBBH. Only genes with at least 10 reads in at least 2 samples were used in the following analysis and low-count genes were removed. The relative gene expression was normalized using the iDEGES method implemented in the TCC package (version 1.2.0) [32]. Transcriptome data of intestine and gill in eel was deposited in DDBJ database with accession number DRA004257 and DRA004258 respectively.

As the histology data suggested major involvement of mucus cells and desquamation of epithelia, we analyzed the known epithelial growth factors and the gene family members in goblet cells [33] to deepen our understanding of the dynamic changes of the epithelia globally. We used *MUC2* and *MUC5AC* as reference markers for the mucus cells. Genes with upregulation, downregulation, and unchanged expression were all included for the protein interaction analysis but gene expression less than 50 reads/million on average were excluded from further analyses.

### Protein interaction network of epithelial growth factors by STRING analysis

The protein interaction network of the known epithelial growth factors in the posterior intestine were extracted from STRING v10 [34] using human as model organism as the database coverage is the highest. A protein interaction networks among genes that expression was upregulated, downregulated, unchanged were constructed by overlapping known interacting partner genes extracted from the database [26].

### Statistical analysis

Plasma Na^+^ concentration, tissue Na content, moisture, and Na concentration, gene expressions in at various times following FW to SW transfer were analyzed by one-way ANOVA followed by Tukey’s multiple-comparison test (GraphPad Prism Ver. 6.0 for Windows, CA, USA). Bound Na contents in the luminal secretions of esophagus and intestine (FW vs. SW 3 h), gene expression between FW to FW (control, 1 h, and 3 h) vs. FW to SW (salinity, 1 h, and 3 h) in gill, anterior and posterior intestine of eels were analyzed by two-way ANOVA, Bonferroni’s test. *p* < 0.05 is considered as significant different among comparisons.

## Results

### Plasma Na^+^ in eels and medaka

Eel plasma Na^+^ did not significantly increase after 3 h (8.9% increase from FW), gradually reached statistical significant after 1 d (24.5% increase from FW), and subsequently established to a lower baseline after 7 d in SW (Fig. 1a). Plasma Na^+^ in medaka significantly increased by 21.1% from 147 ± 2.0 mM to 178 ± 4.8 mM (p < 0.05) 3 h after transfer from FW to 2/3 SW (Table 1) and returned to a new baseline (166 ± 1.6 mM) after 1 d.

### Gene expression and immunohistochemistry of SW-associated transporters in gill and intestine

Na-K-2Cl cotransporter 1α (NKCC1α, *SLC12A2*a) in the gill was gradually upregulated and became significant after 7 d in SW (Fig. 1b). Gill specific *CFTR*b expression was upregulated in a similar delayed fashion as of NKCC1α (Fig. 1c). Na-K-2Cl cotransporter 2β (NKCC2β, *SLC12A1*b) was expressed most abundantly in the anterior and middle intestine among the digestive tract and the upregulation became significant in anterior intestine after 12 h in SW but not in middle intestine. The upregulation reached maximum in 1 to 3 d, and returned to a lower level after 7 d in SW (Fig. 1d).

The immunohistochemistry results of CFTR and NKCC were similar to those of gene expression. Tissue sections from different salinity treatment were mounted on the same slides to ensure identical antibody incubation and color development for comparison. Apical CFTR signals on the ionocytes were observed in after 7 d acclimation in SW (Fig. 2f, box), but were not apparent in the gill of early SW transferred eels (Fig. 2a-e). Basolateral NKCC staining was observed scarcely in the ionocytes in eels from FW to SW 1 d, while increasing intensity and density of NKCC positive ionocytes were observed in SW 3 d and 7 d individuals (Fig. 2g-l). In the anterior intestine, NKCC signals were found on the apical membrane of the mucosal epithelia in all individuals, with an increasing intensity from SW 3 d to SW 7 d (Fig. 2m-r). No apparent localization changes were observed among branchial and intestinal CFTR and NKCC during early SW exposure (SW 3 h to 12 h).

### Sodium and water contents in various tissues

**Fig. 3:**
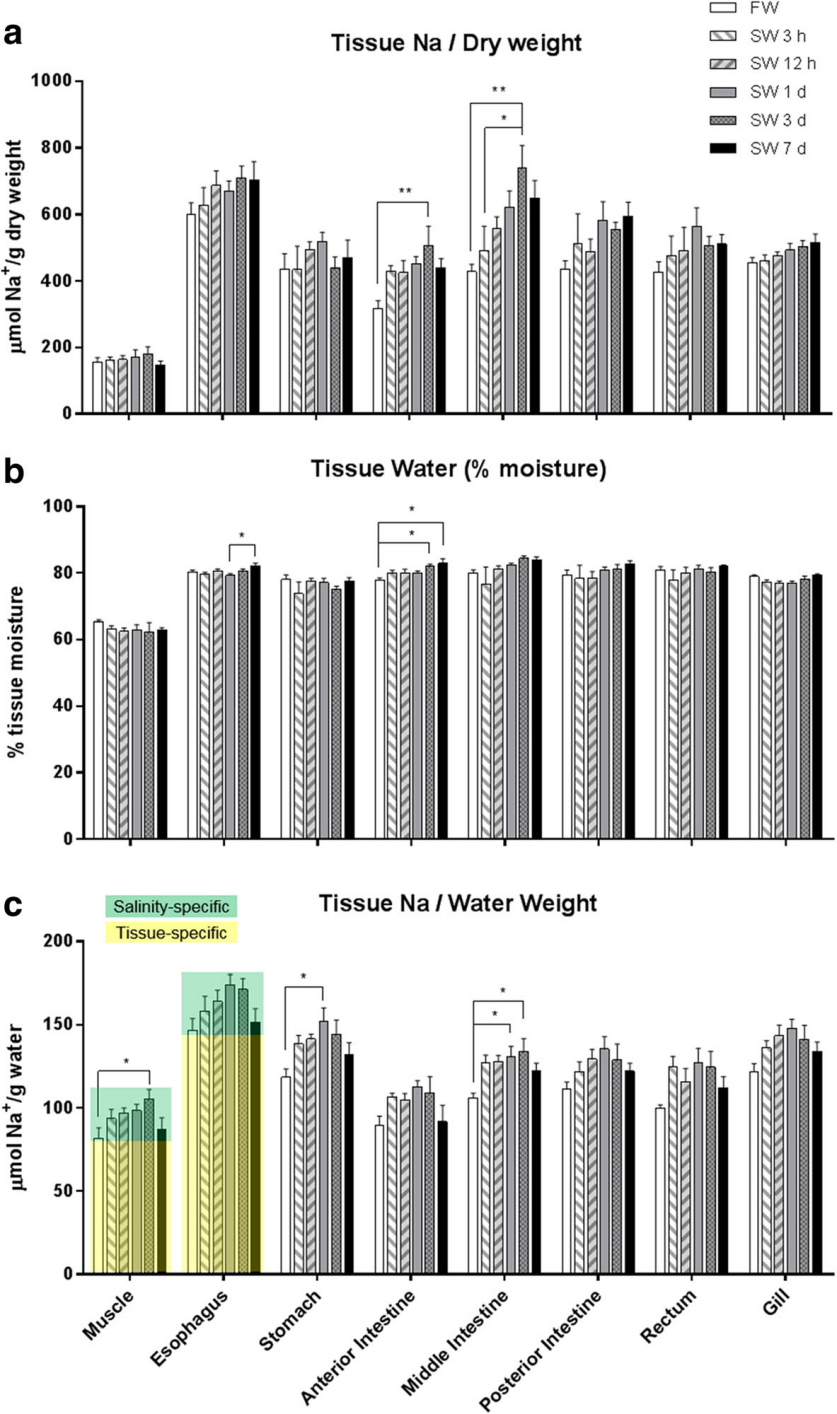
Effects of time-course freshwater (FW) to seawater (SW) transfer on (**a**) tissue Na content, (**b**) tissue moisture, and (**c**) tissue Na concentration in eels. Green and yellow shadings indicates the salinity-specific changes and tissue-specific basal Na concentrations respectively in representative tissues. Statistical differences of salinity transfer (*N* = 6) are indicated by * (*p* < 0.05) and ** (*p* < 0.01) after one way ANOVA, Tukey’s test

As we considered that some organs may store the osmotically inactive Na during the initial phase of SW transfer, we analyzed total Na contents of the gill, skeletal muscle, and digestive tract, which are directly or indirectly exposed to SW. Comparing various tissues examined, the muscle contained the lowest Na, which was not significantly altered by SW transfer (Fig. 3a). The esophagus contained the highest Na content in both FW and SW, which was ~3 folds to that of the muscle. The gill also possessed ~ 2.5 folds higher Na content than muscle. SW transfer increased Na content transiently and most significantly in the middle intestine, and the levels reached the highest level after 3 d and then returned to a lower level toward 7 d in SW.

Muscle moisture was lowest in the skeletal muscle (ca. 65%) compared with those of the gill and digestive tract (ca. 75–80%, Fig. 3b). Generally, the tissue moisture was unaffected by SW transfer compared with the Na content (Fig. 3a). A significant increase in anterior intestine was observed at 3 d and 7 d after SW transfer compared with FW, and between SW 1 d and 7 d in the esophagus. However, the magnitudes of moisture difference were considerably small.

To account for the lower moisture of muscle, Na content was normalized against moisture content to infer the Na concentration among the tissues, with the assumption that all Na was in aqueous form (Fig. 3c). The esophagus possessed the highest basal Na concentration among tissues, which was ~1.5 fold higher to that of the muscle, independent of salinity acclimation. Following esophagus, gill and stomach also contained higher Na concentration compared to muscle. In most tissues examined, the Na concentration increased gradually after SW transfer, reaching a maximum after 3 d, and returned to a lower level after 7 d in SW, a pattern parallel to that of plasma Na^+^. This proportion of the monophasic increase and decrease in Na concentration among different tissues was “salinity-specific” and was due to changes in Na contents in the plasma and ECF, while the higher basal Na concentration was “tissue-specific”, such as those observed in esophagus (Fig. 3c). These tissue-specific Na concentration indicated that those tissues possessed bound Na in non-aqueous state.

### Histological changes after SW transfer in eel gill, skin, and digestive tract

**Fig. 4:**
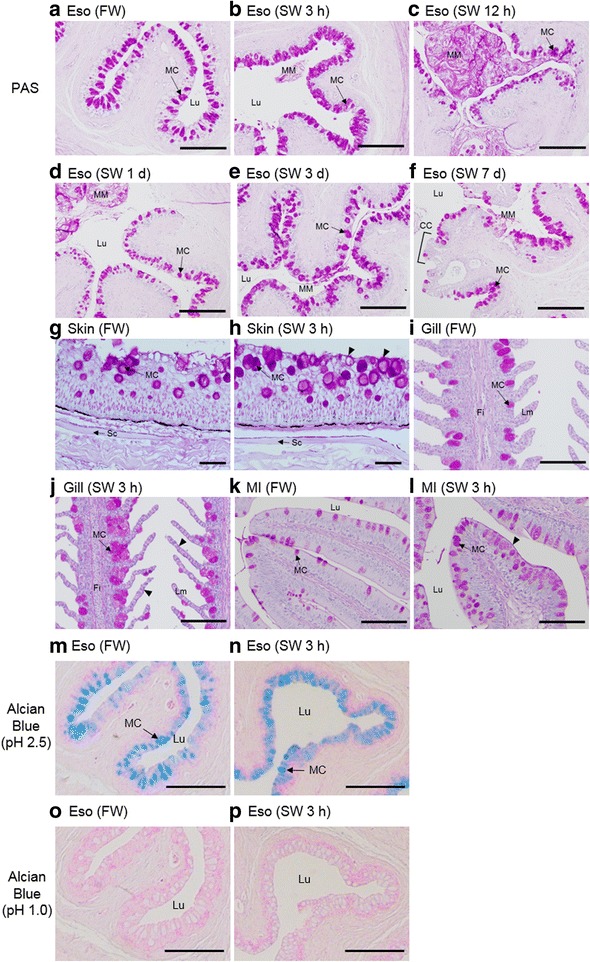
Periodic acid–Schiff (PAS), Alcian blue stainings of esophagus (Eso), skin, gill, and middle intestine (MI) of eels acclimated to FW (**a**, **g**, **i**, **k**, **m**, **o**) or SW after 3 h (**b**, **h**, **j**, **l**, **n**, **p**), 12 h (**c**), 1 d (**d**), 3 d (**e**) 7 d (**f**). PAS- and Alcian blue (pH 2.5)-positive mucus cell (MC) density increased rapidly 3 h after SW transfer in Eso. Alcian blue (pH 1.0) staining was negative in the esophageal epithelia. Mucus mass (MM) with desquamated cells are prominent in the lumen (Lu) during early transfer (from SW 3 h – SW 1 d) in Eso. Absorptive columnar cells (CC) are present on the esophageal villi after 7 d in SW. Arrow heads indicate the mucus layer thickening after SW transfer. Sc = scale; Fi = filament; Lm = lamellae. Scale bar = 200 μm

The time course changes in basic morphology and histology of various osmoregulatory organs exposed to SW were studied to search for any immediate responses during FW to SW transition. In FW esophagus, density of mucus cells with strong PAS-staining was relatively low but increased rapidly after 3 h in SW, and gradually decreased after 12 h, and yet maintained a relatively dense population after 7 d (Fig. 4a-f). Aside from the mucus cell number, we observed increases in mucus secretion and epithelial desquamation during early SW transfer. Absorptive columnar cells developed after 3 d and became more prominent after 7 d in SW (Fig. 4f). Besides the esophagus, a similar increase in mucus cell density was also observed in the gill, skin, and intestine soon after SW exposure (Fig. 4h-m), and the thickness of mucus layers on those tissues also increased. Similar mucus cell distribution in the esophagus were obtained by Alcian blue staining at pH 2.5 (Fig. 4m-n) but not pH 1.0 (Fig. 4o-p), suggesting that sulfated polysaccharides were not major components in the esophageal mucus secretion.

### Bound Na distribution in various tissues by EPMA scan

**Fig. 5:**
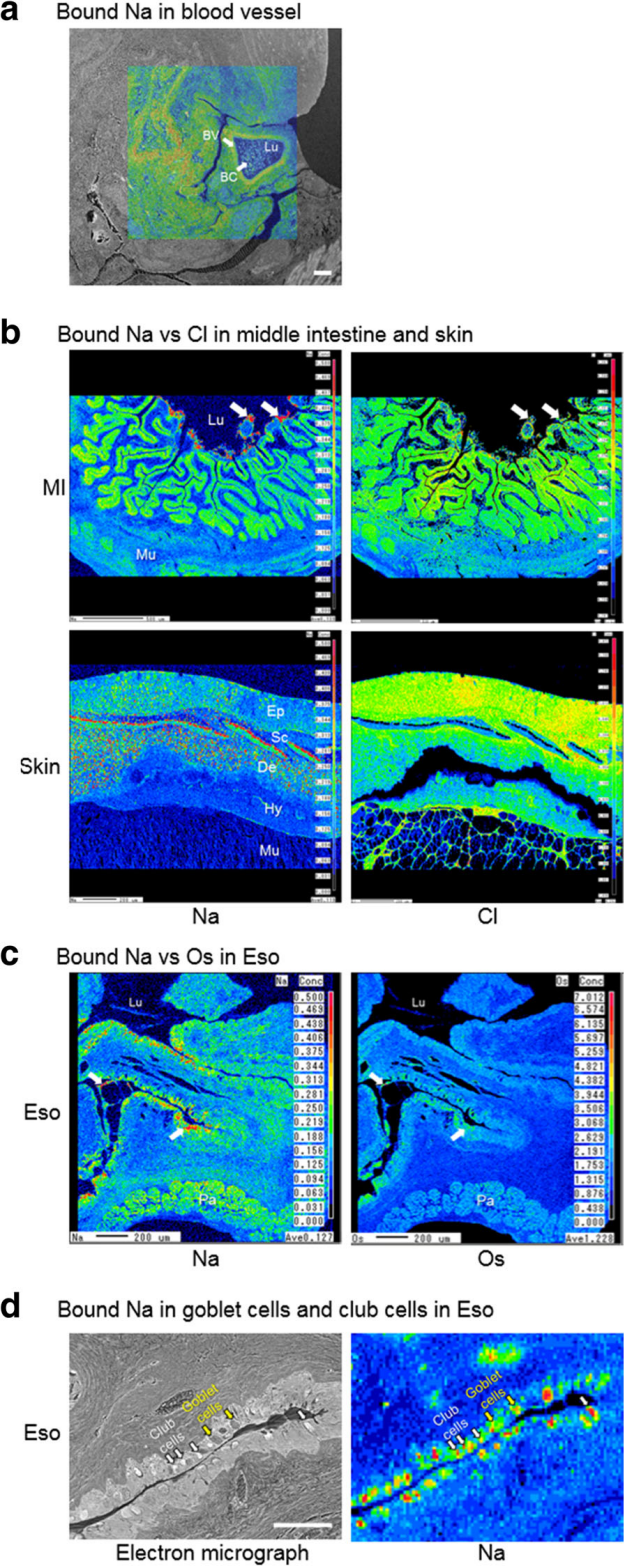
Bound Na, Cl, and Os in eel tissues. **a** Bound Na distribution in a blood vessel (BV) of eel after 1 d in SW. The Na-scan is overlaid on the scanning electron micrograph. The blood vessel lumen is filled with blood cells (BC) with low bound Na in the plasma space, indicating that the fixation process has removed Na from the aqueous space, leaving only bound Na in the tissue. Scale bar = 100 μm. **b** Comparison between bound Na and Cl distribution in the middle intestine and skin after 3 h and 1 d in SW respectively, showing that their distribution are not paralleled. In scale (Sc), bound Na was high while Cl was almost undetectable. The bound Na was higher than the bound Cl in the mucus secretion and desquamated cell mass (while arrow). **c** Comparison between Na and Os distribution in esophagus (Eso) after 3 h and 1 d in SW respectively. The distribution of bound Na and Os were not paralleled. Bound Na was high in mucus cells (white arrows), mucus secretion, and desquamated cell mass with average bound Os. **d** Goblet cells (yellow arrows) and club cells (white arrows) are present on the secretory epithelium of eel esophagus and both cell types contain bound Na. Lu = lumen; Mu = muscle; Pa = pancreas. Lu = lumen; Ep = epidermis; De = dermis; Hy = hypodermis; Mu = muscle

We developed an EPMA method that sufficiently removed aqueous Na^+^ to distinguish bound Na (organic-bound) from free Na^+^ (dissolved). Acetone dehydration eluted the water content, along with the dissolved ions from the samples. The lack of Na signals in the lumen of blood vessels indicated that the Na signals in the EPMA scan are representing bound Na as the plasma contain the highest free Na^+^ (Fig. 5a). Bound Na that remained in smooth muscles situated in deeper region of the specimen, and was at low levels that close to the background Na level of the epoxy resin, indicating that the thickness of the specimen did not affect the dehydration process. Moreover, the Na and Cl patterns by the EPMA scan were different (Fig. 5b), indicating that separate molecules could be involved in manipulating Na^+^ or Cl^−^ at different cellular locations. Unparalleled bound Na and Os was observed in mucus (Fig. 5c), suggesting the molecules with unsaturated carbon chains such as fatty acids are not major candidates for sequestering Na. Besides goblet cells, we identified that club cells (or Clara cells) were also rich in bound Na in the esophagus (Fig. 5d). Club cells were previously described in eel esophagus and they are morphologically distinguishable by their club shape and possessing a large central vacuole [35].

**Fig. 6:**
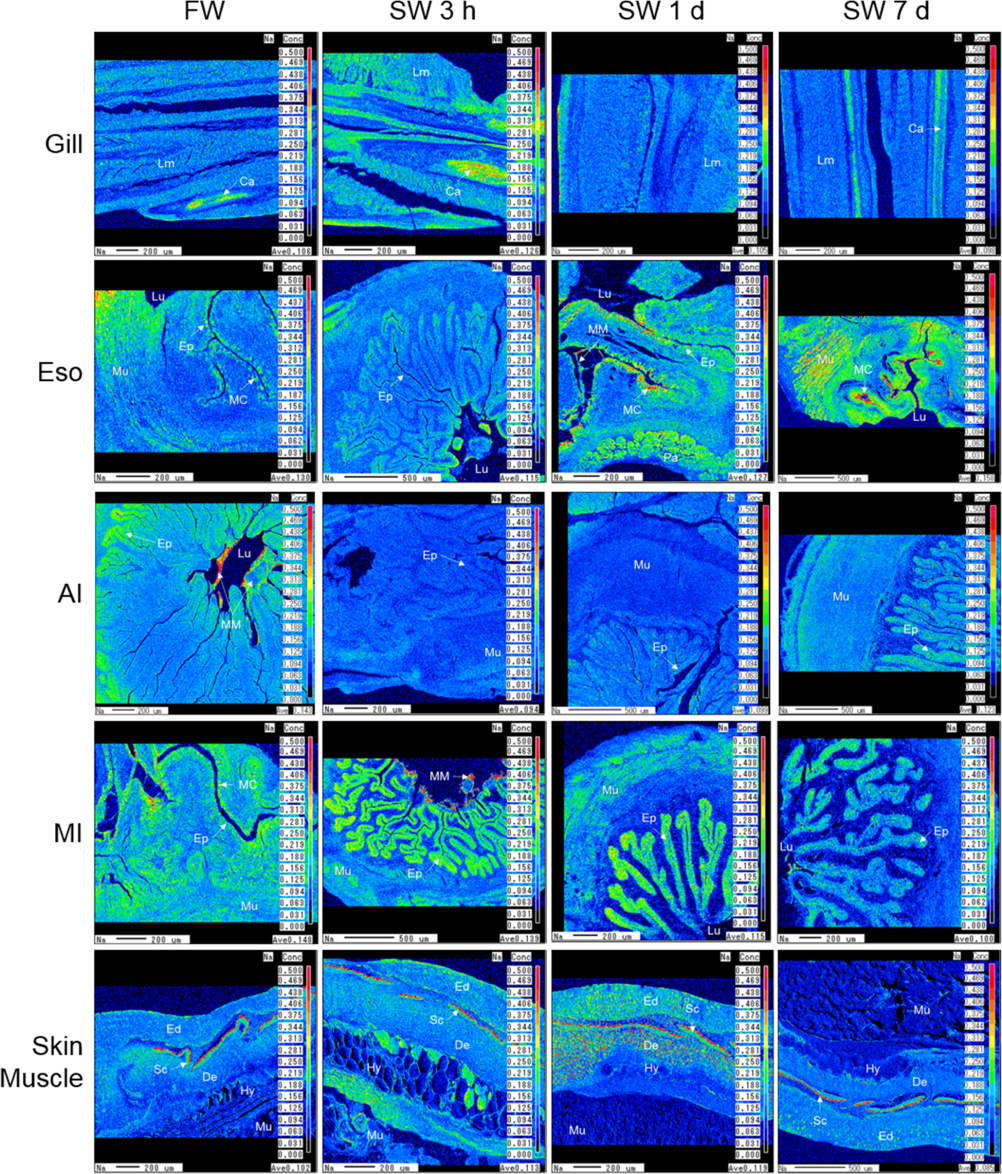
Bound Na distribution in gill, esophagus (Eso), anterior intestine (AI), middle intestine (MI), and skin of eels acclimated to FW, SW 3 h, SW 1 d, or SW 7 d. Scale on the right shows the color gradient of Na concentration. Lm = lamellae; Ca = cartilage; Ep = luminal epithelium; Mu = muscle; MC = mucus cells; MM = mucus mass; Lu = lumen; Pa = pancreas; Ed = epidermis; De = dermis; Hy = hypodermis; Sc = sub-epidermal scale

Although the absolute amount of bound Na cannot be quantified, the relative bound Na distribution can be estimated by the intensity on the EPMA scan maps, which were adjusted to the same scale for comparison among different tissues and regions (Fig. 6). On the gill filament, the bound Na distributed evenly but a larger amount was found in secondary lamellae of the gill 3 h after SW transfer. The cartilage possessed 1.5 fold higher bound Na than the lamellae, but interlamellar regions where ionocytes situated did not show extra accumulation of bound Na. FW esophagus possessed 1.8 fold higher bound Na on the epithelium than the muscle and SW transfer increased the bound Na of mucus cells to 5.1 folds. Bound Na was 5.0 fold higher in discharged mucus mass after 1 d and 7 d in SW. Bound Na signals were 3.0 fold higher in the striated muscle compared to smooth muscle in esophagus after 7 d in SW. In the intestine, the epithelium of villi usually contain higher bound Na but we did not observe bound Na accumulation in the mucus cells. In the anterior intestine of FW eels, bound Na was 2.0 fold higher at the base compared to the tip in villi, and luminal mucus mass possessed 7.0–9.0 fold higher bound Na. Bound Na were 9.2 fold higher in the mucus mass and 1.8 fold higher in the epithelium compared to smooth muscle in the middle intestine 3 h after SW transfer. In the skin, bound Na was 2.0 fold higher at the dermal layer of the skin than in skeletal muscle. The scale in dermis was high in bound Na especially at external side (3.1 folds of muscle), where calcification occurred. In the dermal layer of FW eels, bound Na increased 2.5 folds after 1 d SW transfer, and the bound Na appeared as spot-like scatters. After 7 d in SW, the scattered bound Na decrease to a lesser extent (1.8 fold) in SW condition.

### Gel filtration chromatography

**Fig. 7:**
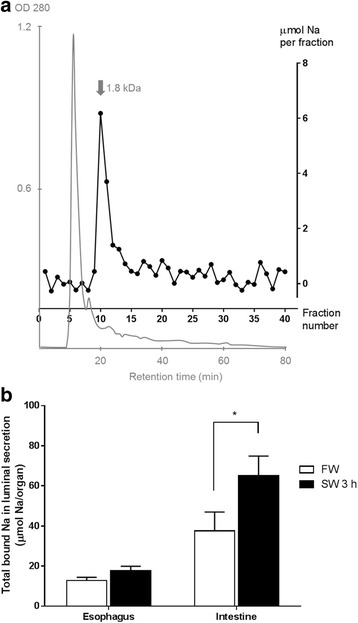
Partial purification of Na-binding molecules from esophageal and intestinal luminal secretion using gel filtration HPLC. **a** A representative chromatograph (OD 280 nm) showing the separation of a FW esophageal luminal secretion sample (grey, left Y-axis). The gel filtration column is equilibrated with 0.1 M Tris-HCl (pH 7.4) at a flowrate of 0.5 mL/min. Large proteins (> 4 kDa) and free Na are eluted at the flow through (10–15 min). The Na content of each 2 min fraction (black, right Y-axis) was superimposed on the chromatograph to indicate the relationship among molecular size, protein quantity, and Na-binding capacities. A bound-Na peak is present with molecular size ~1.8 kDa. **b** Effects of 3 h SW transfer on the total bound Na in the luminal secretions of eel esophagus and intestine (*N* = 5). Total bound Na is defined as the sum of Na contents in non-flow-through fractions in A). Statistical difference between salinity treatment in the same tissue is indicated by * (*p* < 0.05) after two way ANOVA, Bonferroni’s test

Although the EPMA method has prompted the existence of Na-binding molecules visually, it is also technically limited to deduce the quantities of bound Na at physiological range because of the unknown partitioning rate of bound Na during dehydration and embedding. Therefore, we searched for alternative methods to demonstrate the Na-binding capacities in eel secretion more directly. The crude extracts from the luminal secretion of esophagus and intestine were resolved by the gel filtration system with a size exclusion limit of 4 kDa. Most large molecules were eluted in the flow-through (10–15 min) and smaller molecules were eluted sequentially in a decreasing size order (Fig. 7a). The eluted fractions were acidified to release any potential molecule-bound Na, and we observed a Na peak at 20–24 min, indicating that some molecules (~ 1.8 kDa) were sequestering the Na in the secretion. Interestingly, free Na in the flow-through was not detected, indicating that the 1.8 kDa molecules in the secretion have high capacities to capture the existing Na. Total bound Na were observed in both FW and SW 3 h, and a significantly higher level was found in the intestinal secretion in SW 3 h (Fig. 7b), indicating that the mucosal Na-binding molecules sequestered extra Na from drinking during the early SW acclimation.

### Transcriptome search for mucus-cell regulatory genes

**Fig. 8:**
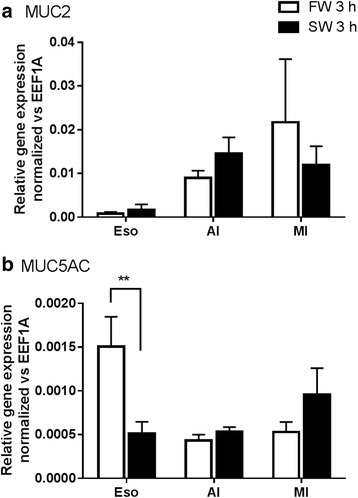
Effects of FW-FW and FW-SW transfer on the mucin gene expressions. **a** *MUC2* and **b** *MUC5AC* in esophagus (Eso), anterior (AI), and middle (MI) intestines (*N* = 5). Statistical difference between salinity transfer in the same tissue is indicated by ** (*p* < 0.01) after two way ANOVA, Bonferroni’s test

**Fig. 9:**
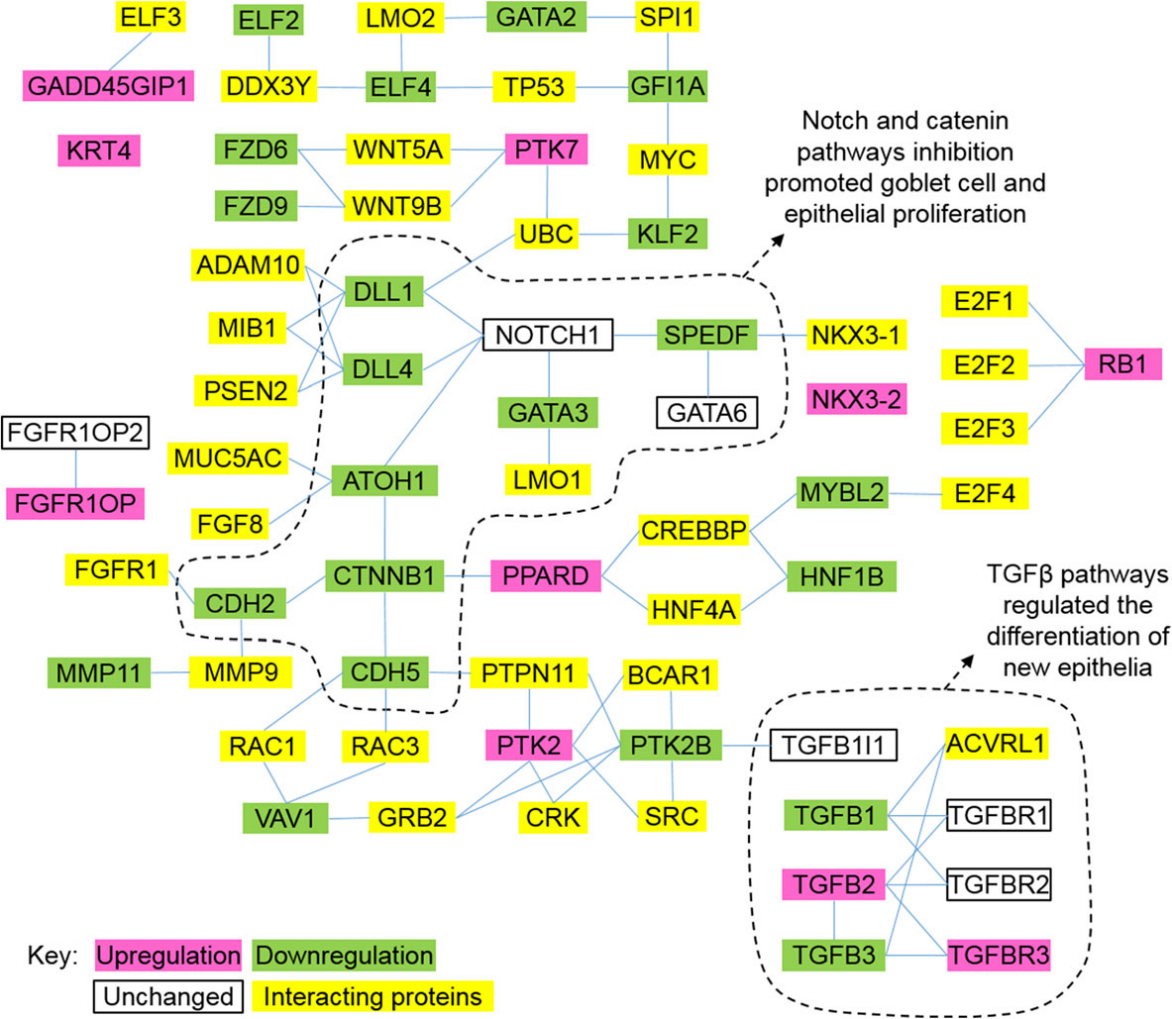
Protein interaction networks of the epithelial growth factors in posterior intestine listed in Additional file 1: Table S1 by STRING analysis. Constitutive and regulatory factors were combined to show the dynamic changes of mucus cells in early SW transfer. Downregulation of notch and β-catenin pathways promoted mucus cell activities, desquamation of epithelial cells, and rapid remodeling of the intestine epithelia

Common gene markers for mucins (*MUC2* and *MUC5AC*) were found but not upregulated in most tissues 3 h post SW transfer (Fig. 8), suggesting that the post-transcriptional factors regulated the mucus cell activities. We examined the expression pattern of reference genes that regulate the epithelial differentiation (Additional file 1: Table S2) using the RNA-seq data and their expressions were categorized into upregulated, downregulated, unchanged. Generally, the number of downregulated genes was higher than that of upregulated genes. *FGFR1OP*, *KRT4*, *PTK2*, *PTK7*, *NKX3–2*, *RB1*, *PPARD*, *TGFB2*, and *TFGBR3* were upregulated during early SW transfer. The integration of protein interaction according to the STRING database among the extracted genes from the posterior intestine transcriptome formed a network that indicates the dynamic changes of both constitutive and regulatory components (Fig. 9). The network suggested that the constitutive *NOTCH1* interacted with downregulated genes including *ATOH1*, *CDH2*, *CDH5*, *CTNNB1, DLL1*, *DLL4*, *GATA3*, and *SPEDF*, leading to an suppression of notch- and catenin-related pathways during early SW transfer. Similarly, the network involving TGFβ was also dynamically affected by SW transfer as shown by the upregulation of *TGFB2* and *TGFBR3*, and downregulation of *TGFB1* and *TGFB3*.

## Discussion

### Where does the Na go? Tissue specific Na storage in eels

Our survey on the plasma ion or osmolality changes in various teleosts revealed a general trend of delayed increase of plasma osmolality in euryhaline species during abrupt FW to SW transfer (Table 1). Stenohaline species such as stream stickleback and goldfish are incapable of blocking the ion influx from SW and died with hypernatremia after SW transfer. Moderately euryhaline species such as medaka can withstand mild hyperosmotic challenge with a maximum increase in plasma Na^+^ 3 h after hyperosmotic challenge, indicating that the ions moved into the body as in those of stenohaline species, but the body rapidly respond to the salt stress by activating of ion-excretion mechanisms [36]. The surprising observation was that most euryhaline teleosts exhibited delayed increases in plasma osmolality after direct SW transfer (Table 1), and we considered that the capability to block the ion influx after abrupt hyperosmotic challenge could be a common feature among euryhaline teleosts. In eels, NKCC and CFTR contents and localization did not markedly change at early SW transfer, suggesting that the transporter-mediated osmoregulatory models derived from the previous studies in SW-acclimated fishes [3] do not contribute to the stabilization of plasma osmolality (Fig. 2).

The present study rather showed unparalleled changes in Na content and moisture, implying that a considerable amount of Na is osmotically inactive (bound Na), and can be stored in eel tissues (Fig. 3). The tissue Na concentration in the muscle is mostly affected by the changes in ECF concentrations in eels. The gradual change of plasma Na^+^ concentration (Fig. 1a) was paralleled to those of tissue Na concentration, and we indicated that portion of changes as “salinity-specific”. This “salinity-specific” portions were highly similar in various tissues including muscle and intestine, indicating that the changes in ECF concentration affect the tissue Na concentration among different parts of the body similarly. However, besides the salinity-specific portion, esophagus possessed c.a. 1.5 fold higher Na concentration, and if all the Na was due to a high extracellular space in esophagus, higher moisture contents in esophagus compared to other tissues such as anterior intestine are expected. It was shown that higher extracellular space in the tissues is associated with higher tissue moisture as well as higher total Na contents because of a high extracellular to intracellular space ratio [37]. However, eels exhibited insignificant regulatory volume increase during SW transfer, as indicated by the relatively constant moisture (c.a. 80%) among gill and various parts of digestive tract (Fig. 3b), thus the high tissue-specific Na concentration in esophagus implies that Na is being sequestered in an alternative space other than extracellular space, an observation that has led to the hypothesis of Na-binding system in the present study.

In Mozambique tilapia, transfer from FW to 27‰ SW increased plasma Na^+^ concentration from c.a. 120 to 190 mM at 3 h and remained at 190 mM at 6 h, but the intracellular Na content increased from c.a. 240 to 350 mmol/kg dcm at 3 h, and to c.a. 650 mmol/kg dcm at 6 h [37]. The net increase in intracellular Na between 3 h and 6 h suggests some systems of Na-sequestering, which cannot be explained alone by the changes in extracellular and intracellular fluid concentrations. The Na-binding concept hypothesized in the present study can address such phenomenon that could not be explained solely by the transporter-mediated osmoregulatory models.

### Activated mucus production in osmoregulatory tissues during SW acclimation

During the initial few hours after SW transfer, the imbibed Na^+^ did not move into the blood stream nor was it stored within the tissues, suggesting that the Na^+^ was trapped in situ on the surface of intestine lumen and gill. With this hypothesis, we investigated the immediate histological changes of these tissues to infer any possible links between the Na binding and histology during early FW to SW transfer. In the esophagus where the imbibed SW was being processed, the early activation of mucus cells and mass production of mucus coincided the hyperosmotic stress from SW exposure and we considered that this could be linked to the observed Na sequestering. An increase in mucus cell numbers correlated to an increase in mucus secretion [38]. As the absorptive columnar cells did not developed until 7 d after SW transfer (Fig. 4f), the esophagus at early phase of SW transfer has limited desalination ability [19, 39, 40]. Desquamated cells appeared in the esophageal lumen between 3 h and 1 d following SW transfer, indicating an active detachment of epithelial cell layers in response to the burst drinking of SW. The mucus and desquamated cells could be a carrier for ions in osmotically inactive form, thus equivalent to salt precipitation that alleviates the osmotic gradient between the imbibed water and blood. Other tissues such as gill and skin, which are directly exposed to SW, increased mucus cell numbers in a similar fashion to that of esophagus, supporting the osmoregulatory role of mucus (Fig. 4g-l).

Nearly a century ago, it was shown that mucus removal from the skin of eels is lethal in SW [41]. Composition, quantity, and ultrastructure of mucus changed according to environmental salinities [42, 43], indicating that eels may produce different types of mucus depending on the environments. Besides surface protection and lubrication, the diffusion gradient of mucus for Cl^−^ was found to be twice as deep as that of Na^+^ in the anterior esophagus of SW eels, suggesting that the mucus contained negative charges that may repel Cl^−^ [43]. The negatively charged mucus is specialized in SW esophagus but not intestine, and could contain the Na-binding molecules that sequester Na^+^ to negate its osmotic potential. In eels, Na^+^ and Cl^−^ fluxes, measured by radioactive isotopes, were not parallel in early FW to SW transition, suggesting that the temporal readjustment of Na-exchange was different from that of Cl-exchange [17]. In frogs, the skin possesses an additional compartment of Na^+^ which was not exchangeable by a hundred fold increase in Na^+^ concentration in the bathing solution [44], indicating bound Na is also present in other vertebrate epithelia. Taken together, the existence of Na-binding molecules in various epithelia and mucus of vertebrates has been implicated in the past electrophysiology and isotopes studies, and we provided further visual evidence to support this in the present study.

### Demonstration of bound Na by EPMA

The gill filament contained more bound Na in scattered forms after 3 h in SW and these Na spots were in agreement with the increased density of mucus cells, suggesting that the Na-binding molecules could be secreted into the mucus to form a micro-film on the surface of gill filaments, where the Na in SW could be trapped to lower the influx.

The esophagus possessed considerable bound Na on the epithelial mucus cells and muscle in both FW and SW, which was in agreement with the higher tissue specific Na storage. Less bound Na was observed in the mucosal epithelia of anterior intestine compared to those of middle intestine, indicating that the production of these Na-binding molecules was region specific, and in agreement with the lower tissue-specific Na concentration in the anterior intestine. The bound Na in the anterior intestine was concentrated at the base of the villi, which was a puzzling phenomenon and yet indicated that special secretory cells are responsible for the production of these molecules. The FW anterior intestine expressed higher *CFTR*a than that of SW and the protein was accumulated in vesicles at the base of the villi [27], suggesting that this region may have different functions and secretory properties, but further studies are required to elucidate the role of Na-binding molecules in this region.

Over five-fold increase in bound Na was identified in the mucus and desquamated cells in the lumen of the middle intestine in 3 h after transfer to SW, indicating that the bound Na in the mucus could stay intact along the digestive tract. This is an important feature for the Na-binding molecules as they should be resistant to digestive enzymes in order to maintain the specific binding in the intestine. In mammals, mucus in the small intestine is a monolayer while double layers of mucus are present in the large intestine with the inner layer being impermeable to luminal bacteria to provide protection [45]. Luminal-epithelial distribution of ions measured by X-ray semi-quantitative analysis in the eel intestinal mucus suggested that it was divided into a layer with high Ca situated towards the center of lumen and a layer with high K and Cl adjacent to the epithelium [46]. Different types of mucus could also be produced by the digestive tract of eels, forming multilayers for functional division including water and nutrient absorption, fluid secretion, Ca-precipitate formation, and Na sequestering.

In the skin, we observed some dense spots of high bound Na in the epidermis and dermis in 1 d after SW transfer, indicating that circulatory forms of Na binding molecules exists, which is also supported by the rich bound Na observed in some striated muscle in the esophagus. The role of this circulatory type bound Na requires further investigation but the implication is that various Na-binding molecules are produced to regulate the Na balance in the ECF in addition to the buffering action in mucus. It is interesting that the sub-epidermal scale that is rich in secondary blood vessels contained high bound Na [47]. With the circulatory Na-binding molecules as carriers, it is also possible that the scale may act as a reserve to provide Na in extreme ion-poor environments such as deionized water [16].

### Partial purification of Na-binding molecules with gel filtration chromatography

To demonstrate the presence of Na-binding molecules in the mucus as proposed by other lines of evidence, we partially purified the Na-binding molecules from the luminal secretion of the digestive tracts using gel filtration chromatography. Addition of Na in all buffers in the purification system was avoided to exploit the intrinsic Na-binding characteristic of the molecules, and all the Na detected was originated from eel esophageal and intestinal secretions. Free Na in crude protein samples should be eluted in the flow through fractions and surprisingly we observed negligible amount of free Na in the extract. Instead, most Na was eluted along with molecules at ~18 kDa, suggesting that small proteins or peptides could be responsible for the Na-binding, but further characterization studies are required to identify the molecules.

Consistently with the results from EPMA, the Na-binding molecules exists in FW eels and considerable bound-Na was measured in the luminal secretion. This supports the possible function of the Na-binding molecules to conserve or extract Na in ion-poor environment such as FW. During early SW transfer, the total bound Na increase, indicating that the Na-binding molecules were sensitive to salinity acclimation, and can sequester the invading Na to lower the osmotic disturbance.

### Interaction of mucus cell-related epithelial growth factors during early SW transfer

We intended to investigate the molecular basis of Na-binding and thus we studied the basic changes in gene expression using our transcriptome databases available in gill, anterior, and posterior intestine in the eels after 1 h – 3 h after SW transfer. Although it was suggested that GAGs are the candidates of the Na-binding molecules in mammals [21, 23, 25], we found that the mucus cells that were rich in bound Na in the esophagus contains insignificant amount of sulfated polysaccharide as shown by Alcian blue staining at pH 1.0 (Fig. 4o-p), suggesting that GAGs may not be the significant components for the Na binding in mucus.

Goblet cell marker genes including *MUC2* and *MUC5AC* did not upregulate soon after SW transfer despite a remarkable activation of mucus production, indicating that the immediate controls for secretory protein production and release are at the level of post-transcriptional regulation [48] to allow a rapid fine-tuning of mucus secretion. Therefore, changes in gene expression as the criteria for screening the transcriptome pool may be insufficient to identify the possible genes that transcribe Na-binding molecules. In addition, our result showed that considerable amount of bound Na also existed in FW, indicating that the gene expression for Na-binding molecules could be constitutive. From the eel transcriptome data, we extracted the expression changes in the known epithelial growth factors that regulate the mucus cell differentiation in mammals [33] to understand how these factors may be integrated into the histological and biochemical changes during early FW to SW transition (Additional file 1: Table S2). Since the Na-binding molecules could be controlled by the same regulatory pathways, the protein interaction network may provide some basic knowledge on the production of Na-binding molecules.

Protein interaction networks supported a dynamic change in mucus cell metabolism and proliferation, and epithelial remodeling (Fig. 9). Inhibition of the Notch pathway as indicated by the downregulation of the ligands *DLL1* and *DLL4*, increased secretory cell differentiation including goblet, enteroendocrine, and Paneth cells [49]. As shown histologically, desquamation of epithelial cells from the digestive tract was accompanied by an increase in mucus secretion, which was also supported by downregulation of the β-catenin (*CTNNB1*) pathway that controls the differentiation of absorptive epithelium. Deletion of β-catenin in mice caused crypt ablation, increased apoptosis, depleted numbers of goblet cells, and detachment of villus absorptive cells from the villus core as intact sheets [50].

Dynamic changes in TGFβ pathways were also prominent during early SW transfer. The TGFβ pathways are important for the differentiation of epithelium in the intestine and recent studies have shown that deletion of *TGFBR2* restricted goblet cell differentiation via repressing *SPDEF* transcription [51]. A marked increase in *TGFBR3* expression was an interesting observation in the development and differentiation of intestinal epithelium as *TGFBR3* regulated the growth of columnar fluid-absorbing cells and mucin-producing goblet cells via modulation of the sensitivities of *TGFB1* and *TGFB2* signaling in mice [52]. In general, the transcriptome data indicated that the regulatory pathways of intestinal differentiation was partially similar to those of mammals, and the expression changes were correlated with the histological changes that occurred at specific time points after SW transfer.

### Integration of Na-binding molecules and transporter-mediated osmoregulation

**Fig. 10:**
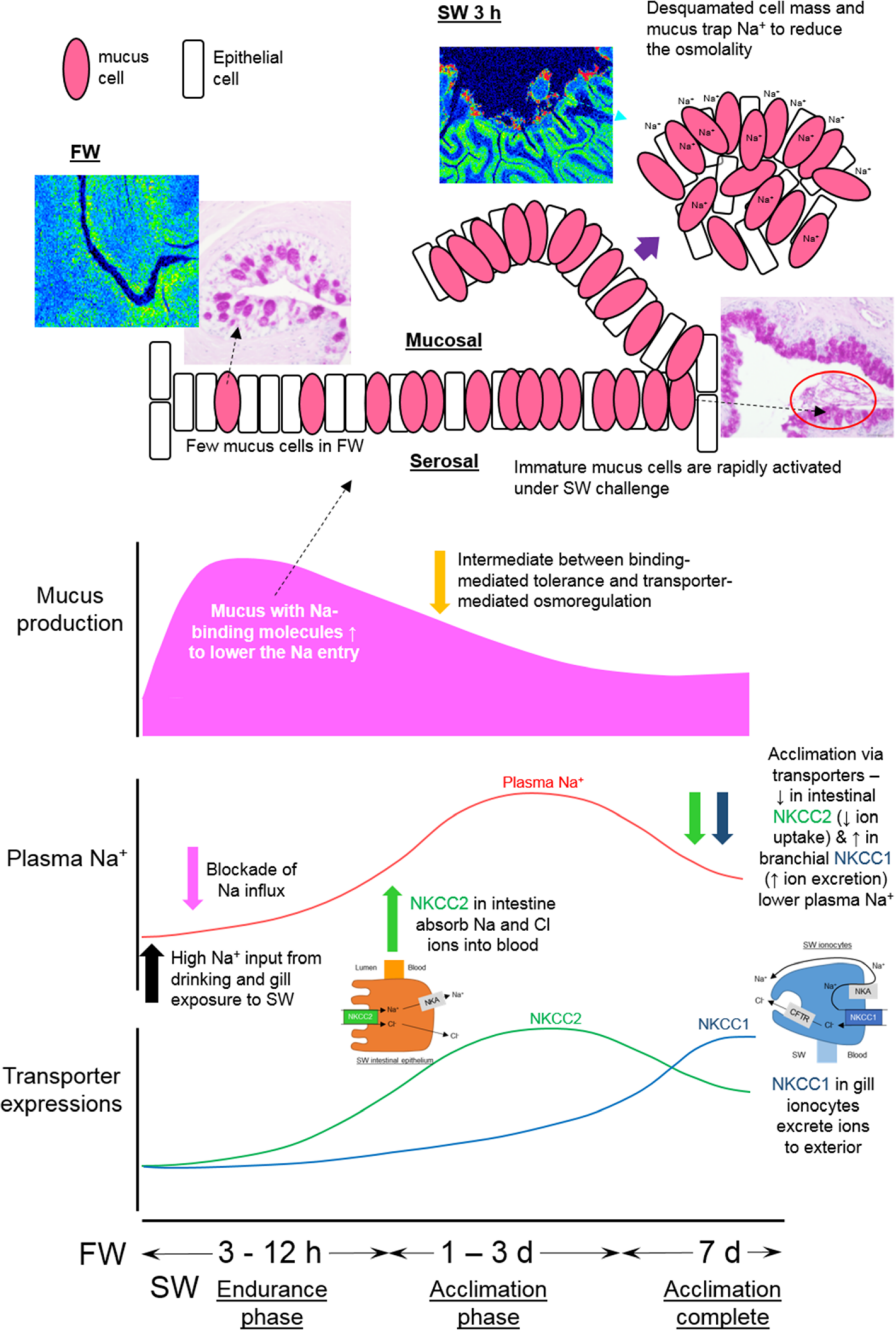
Schematic model of Na trapping by specific binding molecules in mucus and sequential events that leads to the delay biphasic change in plasma Na^+^ in eels following FW to SW transfer. Mucus cells were activated after SW transfer and produced mucus containing Na-binding molecules rapidly, which trapped ionic Na^+^ to reduce the osmotic potential of imbibed SW. The Na-binding and ion absorption/excretion mechanisms were recruited at different time points after SW transfer, and interplayed each other to resolve the salt stress in a specific order, resulting in the signature delay of plasma Na^+^ changes. The circle region indicates desquamated cells containing high bound Na content revealed by EPMA scanning

By integrating the roles of Na binding molecules and known transporter-mediated mechanisms in the gill and digestive tract from literatures, we suggest the sequential events during the FW to SW transition in eels (Fig. 10). Before SW transfer, eels already possess Na-binding molecules in the mucus cells and they could be important for retention of Na in FW. Initial exposure to SW stimulated the eels to drink copiously, which induced massive production of mucus that contains Na-binding molecules to negate the osmotic potential of ionic Na^+^ so as to reduce the osmotic disturbance in the ECF. However, this binding mechanism only provides a temporary buffer to the salt load while the transporting epithelia in various osmoregulatory organs were gradually remodeled from FW- to SW-types. Despite the buffering is temporary, the delay of ion influx into the body is advantageous for the fish to switch the osmoregulatory epithelia gradually. During the acclimation phase when Na-binding and transporter-mediated mechanisms interchanged, the upregulation of intestinal NKCC2β (Fig. 1) and NKA [20] increased water uptake via active absorption of Na^+^ and Cl^−^ [4–6]. The initiation of transporter-mediated osmoregulation resulted in the increase in plasma Na^+^ in SW 1 d, which may induce the production of circulatory type Na-binding molecules that alleviate the adverse effects of high Na^+^ concentration in ECF. Upon the completion of acclimation (3 to 7 d after SW transfer), upregulation of branchial NKCC1α, *CFTR*b, and NKA [19] expression combined to excrete excess ions in ionocytes in eels, returning plasma Na^+^ to a lower level at 7 d [1–3]. The protein contents of intestinal NKCC continued to rise from SW 3 d to 7 d while the NKCC2β expression was maximum at SW 3 d, indicating that the matured transporters accumulated to allow a lower transcription. The interplay between binding-mediated tolerance and transporter-mediated osmoregulation resulted in the signature delayed profile of plasma Na^+^ increase in eels following the FW to SW transfer.

## Conclusion

From an evolutionary perspective, a temporary mechanism to slow down the immediate osmotic stress is advantageous for the estuary and migrating species that are inevitably challenged by salinity fluctuations. Low Na-binding capacity could be one of the limiting factors for the weak euryhaline species such as Japanese medaka that rapidly increased their plasma Na^+^ after SW transfer (Table 1) and thus can only survive a gradual SW transfer. Further species-wide studies will correlate the Na-binding capacities and the euryhalinity among species. In conclusion, we described a binding strategy for osmoregulation, which supplements the transporter-mediated mechanisms. The Na-binding system in teleosts share some similarities to the osmotically inactive skin Na that was correlated to hypertension in human [53], and could provide useful information for the medical studies. We emphasized that the identities of Na-binding molecules in human and fishes were not yet known and these molecules could be among the lists of novel and uncharacterized proteins in the genomes, probably possess high intrinsic negatively charged side chains that can form pocket-like domains to detain cations. Similar biological domains such as Venus-fly trap domain of the extracellular calcium sensing receptor have been shown to hold and transport Ca^2+^, suggesting that it is possible that other cation binding proteins may exist to manipulate Na^+^ [54]. In parallel, we initiated the purification of Na-binding molecules and demonstrated that some 18 kDa molecules in esophageal and intestinal luminal secretions possess high Na-binding capacities. We believe that the integration of Na-binding concept into the field of osmoregulation will broaden researchers’ horizons and resolve many unexplained phenomena.

## Additional file

Additional file 1: Table S2. Summary of expression of known epithelial growth factors that regulates goblet cells (Noah et al., 2011; [33]) during the early seawater transfer (1 h – 3 h) in gill, anterior and posterior intestine of eels. Gene expression was determined by quantitative RNA-seq. Gene expressions were compared between time-dependent FW-FW and FW-SW transfer. Arrows indicate the insignificant change, upregulation (magenta) and downregulation (green). Statistical significance is indicated by *(*p* < 0.05), **(*p* < 0.01), and *** (*p* < 0.001) after two-way ANOVA, Bonferroni’s test. *L.E.* low expression (average less than 50 reads/million), *N.E.* no detectable expression. (DOCX 32 kb)

## Abbreviations

CFTR: Cystic fibrosis transmembrane conductance regulator
ECF: Extracellular fluid
FW: Freshwater
GAG: Glycosaminoglycan
NKA: Na^+^/K^+^-ATPase
NKCC1/NKCC2: Na-K-Cl cotransporter
PAS: Periodic acid / Schiff’s reagent
RBBH: Reciprocal BLAST best hit
SW: Seawater

## References

[1] Hiroi J, McCormick SD (2007). Variation in salinity tolerance, gill Na^+^/K^+^-ATPase, Na^+^/K^+^/2Cl^−^ cotransporter and mitochondria-rich cell distribution in three salmonids *Salvelinus namaycush*, *Salvelinus fontinalis* and *Salmo salar*. J Exp Biol.

[2] Hwang PP, Lee TH, Lin LY (2011). Ion regulation in fish gills: recent progress in the cellular and molecular mechanisms. Am J Physiol Regul Integr Comp Physiol..

[3] Takei Y, Hiroi J, Takahashi H, Sakamoto T (2014). Diverse mechanisms for body fluid regulation in teleost fishes. Am J Physiol Regul Integr Comp Physiol.

[4] Ando M, Mukuda T, Kozaka T (2003). Water metabolism in the eel acclimated to sea water: from mouth to intestine. Comp Biochem Physiol B Biochem Mol Biol..

[5] Cutler CP, Cramb G (2008). Differential expression of absorptive cation-chloride-cotransporters in the intestinal and renal tissues of the European eel (*Anguilla anguilla*). Comp Biochem Physiol B Biochem Mol Biol.

[6] Grosell M (2011). Intestinal anion exchange in marine teleosts is involved in osmoregulation and contributes to the oceanic inorganic carbon cycle. Acta Physiol (Oxf).

[7] Nishimura H, Imai M (1982). Control of renal function in freshwater and marine teleosts. Fed Proc.

[8] Engelund MB, Madsen SS (2011). The role of aquaporins in the kidney of euryhaline teleosts. Front Physiol.

[9] Beyenbach KW (2004). Kidneys sans glomeruli. Am J Physiol Renal Physiol..

[10] Kato A, Muro T, Kimura Y, Li S, Islam Z, Ogoshi M, Doi H, Hirose S (2011). Differential expression of Na^+^-cl^−^ cotransporter and Na^+^-K^+^-cl- cotransporter 2 in the distal nephrons of euryhaline and seawater pufferfishes. Am J Physiol Regul Integr Comp Physiol..

[11] Watanabe T, Takei Y (2011). Molecular physiology and functional morphology of SO_4_^2-^ excretion by the kidney of seawater-adapted eels. J Exp Biol.

[12] Oguri M, Ooshima Y (1977). Early changes in the plasma osmolality and ionic concentrations of rainbow trout and goldfish following direct transfer from fresh-water to sea water. Nippon Suisan Gakk.

[13] Kusakabe M, Ishikawa A, Kitano J (2014). Relaxin-related gene expression differs between anadromous and stream-resident stickleback (*Gasterosteus aculeatus*) following seawater transfer. Gen Comp Endocrinol.

[14] Ogasawara T, Hirano T (1984). Changes in osmotic water permeability of the eel gills during seawater and freshwater adaptation. J Comp Physiol B.

[15] Takei Y, Tsuchida T, Tanakadate A (1998). Evaluation of water intake in seawater adaptation in eel using a synchronized drop counter and pulse injector system. Zoological Sci.

[16] Wong MKS, Takei Y (2012). Changes in plasma angiotensin subtypes in Japanese eel acclimated to various salinities from deionized water to double-strength seawater. Gen Comp Endocrinol.

[17] Kirsch R, Mayer-Gostan N (1973). Kinetics of water and chloride exchanges during adaptation of the European eel to sea water. J Exp Biol.

[18] Takei Y, Okubo J, Yamaguchi K. Effects of cellular dehydration on drinking and plasma angiotensin II level in the eel. *Anguilla japonica* Zoological Sci. 1988;5:43–51.

[19] Takei Y, Wong MKS, Pipil S, Ozaki H, Suzuki Y, Iwasaki W, Kusakabe M (2017). Molecular mechanisms underlying active desalination and low water permeability in the esophagus of eels acclimated to seawater. Am J Physiol Regul Integr Comp Physiol..

[20] Wong MKS, Pipil S, Ozaki H, Suzuki Y, Iwasaki W, Takei Y (2016). Flexible selection of diversified Na^+^/K^+^-ATPase α-subunit isoforms for osmoregulation in teleosts. Zoological Lett.

[21] Titze J (2014). Sodium balance is not just a renal affair. Curr Opin Nephrol Hypertens.

[22] Kopp C, Linz P, Dahlmann A, Hammon M, Jantsch J, Müller DN, Schmieder RE, Cavallaro A, Eckardt KU, Uder M, Luft FC, Titze J (2013). ^23^Na magnetic resonance imaging-determined tissue sodium in healthy subjects and hypertensive patients. Hypertension.

[23] Schafflhuber M, Volpi N, Dahlmann A, Hilgers KF, Maccari F, Dietsch P, Wagner H, Luft FC, Eckardt KU, Titze J (2007). Mobilization of osmotically inactive Na^+^ by growth and by dietary salt restriction in rats. Am J Physiol Renal Physiol.

[24] Jantsch J, Schatz V, Friedrich D, Schröder A, Kopp C, Siegert I, Maronna A, Wendelborn D, Linz P, Binger KJ, Gebhardt M, Heinig M, Neubert P, Fischer F, Teufel S, David JP, Neufert C, Cavallaro A, Rakova N, Küper C, Beck FX, Neuhofer W, Muller DN, Schuler G, Uder M, Bogdan C, Luft FC, Titze J (2015). Cutaneous Na^+^ storage strengthens the antimicrobial barrier function of the skin and boosts macrophage-driven host defense. Cell Metab.

[25] Titze J, Shakibaei M, Schafflhuber M, Schulze-Tanzil G, Porst M, Schwind KH, Dietsch P, Hilgers KF (2004). Glycosaminoglycan polymerization may enable osmotically inactive Na^+^ storage in the skin. Am J Physiol Heart Circ Physiol.

[26] Wong MKS, Ozaki H, Suzuki Y, Iwasaki W, Takei Y (2014). Discovery of transcription factors for seawater acclimation in fish intestine via a transcriptomic approach. BMC Genomics.

[27] Wong MKS, Pipil S, Kato A, Takei Y, Duplicated CFTR (2016). Isoforms in eels diverged in regulatory structures and osmoregulatory functions. Comp Biochem Physiol A Mol Integr Physiol..

[28] Henkel CV, Dirks RP, de Wijze DL, Minegishi Y, Aoyama J, Jansen HJ, Turner B, Knudsen B, Bundgaard M, Hvam KL, Boetzer M, Pirovano W, Weltzien FA, Dufour S, Tsukamoto K, Spaink HP, van den Thillart GE (2012). First draft genome sequence of the Japanese eel, *Anguilla japonica*. Gene.

[29] Kim D, Pertea G, Trapnell C, Pimentel H, Kelley R, Salzberg SL (2013). TopHat2: accurate alignment of transcriptomes in the presence of insertions, deletions and gene fusions. Genome Biol.

[30] Rice P, Longden I, Bleasby AEMBOSS (2000). The European molecular biology open software suite. Trends Genet.

[31] Camacho C, Coulouris G, Avagyan V, Ma N, Papadopoulos J, Bealer K, Madden TL (2009). BLAST+: architecture and applications. BMC Bioinformatics..

[32] Sun J, Nishiyama T, Shimizu K, Kadota K (2013). TCC: an R package for comparing tag count data with robust normalization strategies. BMC Bioinformatics.

[33] Noah TK, Donahue B, Shroyer NF (2011). Intestinal development and differentiation. Exp Cell Res.

[34] Szklarczyk D, Franceschini A, Wyder S, Forslund K, Heller D, Huerta-Cepas J, Simonovic M, Roth A, Santos A, Tsafou KP, Kuhn M, Bork P, Jensen LJ, von Mering C (2015). STRING v10: protein-protein interaction networks, integrated over the tree of life. Nucleic Acids Res.

[35] Nakamura O, Watanabe T, Kamiya H, Muramoto K (2001). Galectin containing cells in the skin and mucosal tissues in Japanese conger eel, *Conger myriaster*: an immunohistochemical study. Dev Comp Immunol.

[36] Shen WP, Horng JL, Lin LY (2011). Functional plasticity of mitochondrion-rich cells in the skin of euryhaline medaka larvae (*Oryzias latipes*) subjected to salinity changes. Am J Physiol Regul Integr Comp Physiol.

[37] Assem H, Hanke W (1978). Volume regulation of muscle cells in the euryhaline teleost, *Tilapia mossambica*. Comp Biochem Physiol.

[38] Okada SF, Zhang L, Kreda SM, Abdullah LH, Davis CW, Pickles RJ, Lazarowski ER, Boucher RC (2011). Coupled nucleotide and mucin hypersecretion from goblet-cell metaplastic human airway epithelium. Am J Respir Cell Mol Biol.

[39] Yamamoto M, Hirano T (1978). Morphological changes in the esophageal epithelium of the eel, *Anguilla japonica*, during adaptation to seawater. Cell Tissue Res.

[40] Nagashima K, Ando M (1994). Characterization of esophageal desalination in the seawater eel, *Anguilla japonica*. J Comp Physiol B.

[41] Portier P, Duval M (1922). Variations de la pression osmotique du sang de l'anguille essuyée en fonction des modifications de la salinité du milieu exterieur. CR Acad Sci Paris. French.

[42] Lemoine AM, Olivereau M (1973). Variations de la teneur en acide N-acetyl-neuraminique de l'intestin de l'anguille, au cours des changements de salinité. CR Soc Biol.

[43] Simonneaux V, Barra JA, Humbert W, Kirsch R (1987). The role of mucus in ion absorption by the oesophagus of sea-water eel (*Anguilla anguilla* L.) electrophysiological, structural and cytochemical investigations. J Comp Physiol B.

[44] Cereijido M, Reisin I, Rotunno CA (1968). The effect of sodium concentration on the content and distribution of sodium in the frog skin. J Physiol.

[45] Johansson ME, Ambort D, Pelaseyed T, Schütte A, Gustafsson JK, Ermund A, Subramani DB, Holmén-Larsson JM, Thomsson KA, Bergström JH, van der Post S, Rodriguez-Piñeiro AM, Sjövall H, Bäckström M, Hansson GC (2011). Composition and functional role of the mucus layers in the intestine. Cell Mol Life Sci.

[46] Humbert W, Kirsch R, Simonneaux VI (1986). Mucus involved in biocrystallization? Study of the intestinal mucus of the sea-water eel *Anguilla anguilla* L. Cell Tissue Res.

[47] Tyszkiewicz K (1969). Structure and vascularization of the skin of the pike (*Esox lucius* L.). Acta Biol Crac Zool.

[48] Ammit AJ (2005). The role of mRNA stability in airway remodelling. Pulm Pharmacol Ther.

[49] VanDussen KL, Carulli AJ, Keeley TM, Patel SR, Puthoff BJ, Magness ST, Tran IT, Maillard I, Siebel C, Kolterud Å, Grosse AS, Gumucio DL, Ernst SA, Tsai YH, Dempsey PJ, Samuelson LC (2012). Notch signaling modulates proliferation and differentiation of intestinal crypt base columnar stem cells. Development.

[50] Ireland H, Kemp R, Houghton C, Howard L, Clarke AR, Sansom OJ, Winton DJ (2004). Inducible Cre-mediated control of gene expression in the murine gastrointestinal tract: effect of loss of beta-catenin. Gastroenterology.

[51] McCauley HA, Liu CY, Attia AC, Wikenheiser-Brokamp KA, Zhang Y, Whitsett JA, Guasch G (2014). TGFβ signaling inhibits goblet cell differentiation via SPDEF in conjunctival epithelium. Development.

[52] Deng X, Bellis S, Yan Z, Friedman E (1999). Differential responsiveness to autocrine and exogenous transforming growth factor (TGF) beta1 in cells with nonfunctional TGF-beta receptor type III. Cell Growth Differ.

[53] Kopp C, Linz P, Wachsmuth L, Dahlmann A, Horbach T, Schöfl C, Renz W, Santoro D, Niendorf T, Müller DN, Neininger M, Cavallaro A, Eckardt KU, Schmieder RE, Luft FC, Uder M, Titze J (2012). (23)Na magnetic resonance imaging of tissue sodium. Hypertension.

[54] Herberger AL, Loretz CA (2013). Vertebrate extracellular calcium-sensing receptor evolution: selection in relation to life history and habitat. Comp Biochem Physiol Part D Genomics Proteomics.

[55] Kammerer BD, Cech JJ, Kültz D (2010). Rapid changes in plasma cortisol, osmolality, and respiration in response to salinity stress in tilapia (*Oreochromis mossambicus*). Comp Biochem Physiol A Mol Integr Physiol.

[56] Wang PJ, Lin CH, Hwang LY, Huang CL, Lee TH, Hwang PP (2009). Differential responses in gills of euryhaline tilapia, *Oreochromis mossambicus*, to various hyperosmotic shocks. Comp Biochem Physiol A Mol Integr Physiol..

[57] Velan A, Hulata G, Ron M, Cnaani A (2011). Comparative time-course study on pituitary and branchial response to salinity challenge in Mozambique tilapia (*Oreochromis mossambicus*) and Nile tilapia (*O. niloticus*). Fish Physiol Biochem.

[58] Fontaínhas-Fernandes A, Gomes EF, Reis-Henriques MA, Coimbra J (2003). Effect of cortisol on some osmoregulatory parameters of the teleost, *Oreochromis niloticus* L, after transference from freshwater to seawater. Arq Bras Med Vet Zootec.

[59] Tipsmark CK, Kiilerich P, Nilsen TO, Ebbesson LO, Stefansson SO, Madsen SS (2008). Branchial expression patterns of claudin isoforms in Atlantic salmon during seawater acclimation and smoltification. Am J Physiol Regul Integr Comp Physiol.

[60] Singer TD, Tucker SJ, Marshall WS, Higgins CFA (1998). Divergent CFTR homologue: highly regulated salt transport in the euryhaline teleost *F. heteroclitus*. Am J Phys.

[61] Marshall WS, Emberley TR, Singer TD, Bryson SE, Mccormick SD (1999). Time course of salinity adaptation in a strongly euryhaline estuarine teleost, *Fundulus heteroclitus*: a multivariable approach. J Exp Biol.

