# Supplementary material for: A sodium binding system alleviates acute salt stress during seawater acclimation in eels
Source: Zoological Lett. 2017 Dec 12;3:22. doi: 10.1186/s40851-017-0081-8 (PMC5727781; doi:10.1186/s40851-017-0081-8)
Supplement: Additional file 1: Table S2. — Summary of expression of known epithelial growth factors that regulates goblet cells (Noah et al., 2011; [33]) during the early seawater transfer (1 h – 3 h) in gill, anterior and posterior intestine of eels. Gene expression was determined by quantitative RNA-seq. Gene expressions were compared between time-dependent FW-FW and FW-SW transfer. Arrows indicate the insignificant change, upregulation (magenta) and downregulation (green). Statistical significance is indicated by *(p < 0.05), **(p < 0.01), and *** (p < 0.001) after two-way ANOVA, Bonferroni’s test. L.E. low expression (average less than 50 reads/million), N.E. no detectable expression. (DOCX 32 kb) [file 40851_2017_81_MOESM1_ESM.docx]

| Gene symbol | Gene name | Gill | |  | Anterior intestine | |  | Posterior intestine | |
| --- | --- | --- | --- | --- | --- | --- | --- | --- | --- |
|  |  | 1 h | 3 h |  | 1 h | 3 h |  | 1 h | 3 h |
| APC | Adenomatous Polyposis Coli | → | → |  | → | → |  | → | → |
| ATOH1 | Atonal BHLH Transcription Factor 1 | N.E. | N.E. |  | ↓** | ↓* |  | ↓*** | ↓*** |
| CDH2 | Cadherin 2 | L.E. | L.E. |  | → | → |  | → | ↓** |
| CDH5 | Cadherin 5 | → | → |  | → | → |  | ↓*** | ↓* |
| CDH8 | Cadherin 8 | → | → |  | → | → |  | → | → |
| CDH17 | Cadherin 17 | L.E. | L.E. |  | → | → |  | → | → |
| CDH19 | Cadherin 19 | → | → |  | L.E. | L.E. |  | L.E. | L.E. |
| CDHR2 | Cadherin Related Family Member 2 | L.E. | L.E. |  | → | → |  | → | → |
| CDX4 | Caudal Type Homeobox 4 | N.E. | N.E. |  | N.E. | N.E. |  | L.E. | L.E. |
| CTTNB1 | Catenin Beta 1 | → | → |  | ↓* | → |  | ↓* | → |
| DLL1 | Delta-Like 1 | L.E. | L.E. |  | → | ↓** |  | → | ↓*** |
| DLL4 | Delta-Like 4 | → | → |  | → | → |  | ↓*** | ↓** |
| EGFRa | Epidermal Growth Factor Receptor | → | → |  | → | ↑* |  | → | → |
| ELF2 | E74 Like ETS Transcription Factor 2 | → | → |  | → | → |  | ↓** | ↓* |
| ELF4 | E74 Like ETS Transcription Factor 4 | → | → |  | → | → |  | → | ↓* |
| FGF2 | Fibroblast Growth Factor 2 | L.E. | L.E. |  | L.E. | L.E. |  | L.E. | L.E. |
| FGF7 | Fibroblast Growth Factor 7 | L.E. | L.E. |  | N.E. | N.E. |  | L.E. | L.E. |
| FGF12 | Fibroblast Growth Factor 12 | L.E. | L.E. |  | L.E. | L.E. |  | L.E. | L.E. |
| FGF16 | Fibroblast Growth Factor 16 | L.E. | L.E. |  | L.E. | L.E. |  | → | → |
| FGF18 | Fibroblast Growth Factor 18 | L.E. | L.E. |  | N.E. | N.E. |  | L.E. | L.E. |
| FGF19 | Fibroblast Growth Factor 19 | N.E. | N.E. |  | → | → |  | → | → |
| FGFR1OP | FGFR1 Oncogene Partner | → | → |  | ↑* | ↑* |  | → | ↑* |
| FGFR1OP2 | FGFR1 Oncogene Partner 2 | → | → |  | → | → |  | → | → |
| FGFRL1 | Fibroblast Growth Factor Receptor-Like 1 | → | → |  | → | → |  | → | → |
| FN1 | Fibronectin 1 | → | → |  | → | → |  | → | → |
| FOXA3 | Forkhead Box A3 | → | → |  | → | → |  | → | → |
| FZD2 | Frizzled Class Receptor 2 | → | → |  | → | ↓** |  | → | → |
| FZD4 | Frizzled Class Receptor 4 | L.E. | L.E. |  | L.E. | L.E. |  | L.E. | L.E. |
| FZD6 | Frizzled Class Receptor 6 | → | → |  | → | ↓* |  | → | ↓** |
| FZD7 | Frizzled Class Receptor 7 | → | → |  | → | ↓* |  | → | → |
| FZD8a | Frizzled Class Receptor 8 | → | → |  | L.E. | L.E. |  | L.E. | L.E. |
| FZD9 | Frizzled Class Receptor 9 | → | → |  | L.E. | L.E. |  | → | ↓** |
| FZD10 | Frizzled Class Receptor 10 | → | → |  | L.E. | L.E. |  | L.E. | L.E. |
| GADD45GIP1 | GADD45G Interacting Protein 1 | → | → |  | → | → |  | → | ↑* |
| GATA1 | GATA Binding Protein 1 | ↑** | ↓* |  | L.E. | L.E. |  | L.E. | L.E. |
| GATA2a | GATA Binding Protein 2 | → | → |  | → | → |  | ↓* | → |
| GATA3 | GATA Binding Protein 3 | → | ↓* |  | → | ↓* |  | ↓*** | ↓*** |
| GATA4 | GATA Binding Protein 4 | N.E. | N.E. |  | → | → |  | L.E. | L.E. |
| GATA5 | GATA Binding Protein 5 | N.E. | N.E. |  | → | → |  | → | → |
| GATA6 | GATA Binding Protein 6 | → | → |  | → | → |  | → | → |
| GFI1Aa | Growth Factor Independent 1 Transcription Repressor | → | → |  | → | → |  | ↓** | ↓*** |
| HES1 | Hes Family BHLH Transcription Factor 1 | → | → |  | → | → |  | → | → |
| HNF1B | HNF1 Homeobox B | N.E. | N.E. |  | → | → |  | ↓* | ↓*** |
| KLF2 | Kruppel-Like Factor 2 | → | → |  | → | → |  | ↓** | → |
| KLF3 | Kruppel-Like Factor 3 | → | → |  | → | → |  | → | → |
| KLF4 | Kruppel-Like Factor 4 | → | → |  | → | ↑* |  | → | → |
| KLF6 | Kruppel-Like Factor 6 | → | → |  | → | → |  | → | → |
| KLF7b | Kruppel-Like Factor 7 | → | → |  | L.E. | L.E. |  | L.E. | L.E. |
| KLF11b | Kruppel-Like Factor 11 | → | → |  | → | → |  | → | → |
| KLF12 | Kruppel-Like Factor 12 | L.E. | L.E. |  | L.E. | L.E. |  | L.E. | L.E. |
| KLF13 | Kruppel-Like Factor 13 | → | ↑** |  | → | ↑* |  | → | → |
| KLF14 | Kruppel-Like Factor 14 | → | → |  | → | ↑* |  | → | → |
| KLF15 | Kruppel-Like Factor 15 | L.E. | L.E. |  | L.E. | L.E. |  | L.E. | L.E. |
| KRT4 | Keratin 4 | → | → |  | → | → |  | → | ↑* |
| KRT9 | Keratin 9 | → | → |  | L.E. | L.E. |  | → | → |
| LGR4 | Leucine-Rich Repeat Containing G Protein-Coupled Receptor 4 | → | → |  | L.E. | L.E. |  | L.E. | L.E. |
| MSI2 | Musashi RNA Binding Protein 2 | → | → |  | → | → |  | → | → |
| MMP11a | Matrix Metallopeptidase 11 | → | → |  | → | → |  | ↓** | ↓** |
| MMP13 | Matrix Metallopeptidase 13 | → | → |  | → | → |  | → | → |
| MMP17 | Matrix Metallopeptidase 17 | L.E. | L.E. |  | L.E. | L.E. |  | L.E. | L.E. |
| MMP19 | Matrix Metallopeptidase 19 | → | → |  | → | → |  | → | → |
| MMP23B | Matrix Metallopeptidase 23B | → | → |  | L.E. | L.E. |  | L.E. | L.E. |
| MYBL2 | MYB Proto-Oncogene Like 2 | → | → |  | → | → |  | → | ↓** |
| MYC | V-Myc Avian Myelocytomatosis Viral Oncogene Homolog | → | → |  | → | → |  | → | → |
| NEUROD1 | Neuronal Differentiation 1 | N.E. | N.E. |  | → | → |  | → | → |
| NKX2-2 | NK2 Homeobox 2 | N.E. | N.E. |  | L.E. | L.E. |  | L.E. | L.E. |
| NKX3-2 | NK3 Homeobox 2 | → | → |  | → | → |  | → | ↑* |
| NOTCH1a | Notch 1 | → | → |  | → | → |  | → | → |
| NOX1 | NADPH Oxidase 1 | → | → |  | → | ↑* |  | → | → |
| NOX4 | NADPH Oxidase 4 | → | → |  | → | → |  | → | → |
| PAX6 | Paired Box 6 | N.E. | N.E. |  | → | → |  | L.E. | L.E. |
| PDX1 | Pancreatic And Duodenal Homeobox 1 | L.E. | L.E. |  | → | → |  | L.E. | L.E. |
| PPARD | Peroxisome Proliferator Activated Receptor Delta | ↓* | → |  | ↑** | → |  | ↑* | → |
| PTK2 | Protein Tyrosine Kinase 2 | → | → |  | → | → |  | ↑* | → |
| PTK2Bb | Protein Tyrosine Kinase 2 Beta | → | → |  | → | → |  | → | ↓** |
| PTK6 | Protein Tyrosine Kinase 6 | → | ↓** |  | → | → |  | → | → |
| PTK7 | Protein Tyrosine Kinase 7 | → | → |  | ↑** | ↑* |  | ↑* | → |
| RB1 | Retinoblastoma 1 | → | → |  | N.E. | N.E. |  | → | ↓** |
| SPDEF | SAM Pointed Domain Containing ETS Transcription Factor | N.E. | N.E. |  | → | → |  | ↓*** | ↓*** |
| STK11 | Serine/Threonine Kinase 11 | → | → |  | L.E. | L.E. |  | L.E. | L.E. |
| STK11IP | Serine/Threonine Kinase 11 Interacting Protein | → | → |  | → | → |  | → | → |
| TGFB1 | Transforming Growth Factor Beta 1 | → | → |  | → | → |  | ↓* | ↓** |
| TGFB1I1 | Transforming Growth Factor Beta 1 Induced Transcript 1 | → | → |  | → | → |  | → | → |
| TGFB2 | Transforming Growth Factor Beta 2 | → | → |  | → | → |  | ↑** | ↑** |
| TGFB3 | Transforming Growth Factor Beta 3 | → | → |  | ↓** | ↓** |  | ↓* | → |
| TGFBI | Transforming Growth Factor Beta Induced | → | → |  | → | → |  | → | → |
| TGFBR2 | Transforming Growth Factor Beta Receptor 2 | → | → |  | L.E. | L.E. |  | L.E. | L.E. |
| TGFBR3 | Transforming Growth Factor Beta Receptor 3 | → | → |  | → | ↑* |  | → | ↑** |
| TGFBRAP1 | Transforming Growth Factor Beta Receptor Associated Protein 1 | → | → |  | L.E. | L.E. |  | L.E. | L.E. |
| VAV1 | Vav Guanine Nucleotide Exchange Factor 1 | → | → |  | → | → |  | ↓* | ↓*** |
|  |  |  |  |  |  |  |  |  |  |
